# Synthesis of Trifluoromethylated Analogues of the Cyclic Lipopeptide Iturin A and Evaluation of their Antifungal Activity

**DOI:** 10.1002/cplu.202500590

**Published:** 2025-12-12

**Authors:** Periklis Karamanis, Matthew Kiernan, Jimmy Muldoon, Paul Evans, Cormac D. Murphy, Marina Rubini

**Affiliations:** ^1^ UCD School of Chemistry University College Dublin Dublin Ireland; ^2^ BiOrbic Bioeconomy SFI Research Centre University College Dublin Dublin Ireland; ^3^ UCD School of Biomolecular and Biomedical Science University College Dublin Dublin Ireland; ^4^ Conway Institute for Biomolecular and Biomedical Research University College Dublin Dublin Ireland

**Keywords:** antifungal agents, cyclic lipopeptides, iturin A, late‐stage trifluoromethylation, peptide cyclisation, peptides, trifluoromethylated peptides

## Abstract

The rise of antifungal resistance threatens public health and agriculture. Iturin A, a cyclic lipopeptide produced by *Bacillus* species, is known for its antifungal activity against various pathogens. In this study, three novel trifluoromethylated analogues of iturin A were synthesised as potential ^19^F NMR probes and to compare their bioactivity with the natural compound. Trifluoromethylation targeted the D‐tyrosine and iturinic acid residues, which are critical for antifungal activity. Fluorinated building blocks were prepared via oxidative radical trifluoromethylation for D‐tyrosine and, notably, via electrophilic trifluoromethylation combined with a chiral auxiliary‐based approach for the iturinic acid, marking the first synthesis of a terminally trifluoromethylated long‐chain β‐amino fatty acid. Peptide assembly was achieved through solid‐phase synthesis followed by on‐resin cyclisation, alongside a high‐yielding late‐stage aromatic trifluoromethylation method. Bioactivity assays revealed that the mono‐trifluoromethylated tyrosine analogue exhibited slight activity loss against *Candida albicans* and greater loss against *Fusarium graminearum*. The bis‐trifluoromethylated tyrosine analogue lost activity against both fungi, while the alkyl‐trifluoromethylated analogue retained full activity against *C. albicans* and showed a minor activity loss against *F. graminearum*. These analogues provide insights into site‐specific trifluoromethylation effects, can serve as valuable ^19^F NMR probes, and can be a platform for further iturin A analogue development.

## Graphical abstract

Iturin A is an antifungal lipopeptide with broad applications. Here, we present a novel synthesis and semisynthesis of trifluoromethylated iturin A analogues. Their antifungal activity was evaluated against *Fusarium graminearum* and *Candida albicans*. The lipopeptides are expected to be useful ^19^F NMR probes for mechanistic studies and the proposed synthesis can enable the development of further iturin A analogues.

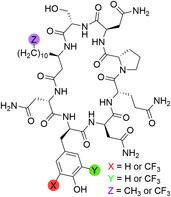

## 1 Introduction

The growing antifungal resistance crisis, paired with the limited arsenal of available drug options, poses a serious threat to health, agriculture and food safety worldwide [1, 2]. This global threat underscores the urgent need not only to develop new antifungal compounds, but also to elucidate the mechanisms of action of existing agents to guide future drug design. Soil bacteria, such as *Bacillus* spp., produce a wide array of secondary metabolites with pronounced antifungal activity [3]. In fact, many *Bacillus* species, including *B. subtilis* and *B. velezensis*, are used as microbial biocontrol agents in the agricultural sector (e.g., Serenade) [4].

**FIGURE 1:**
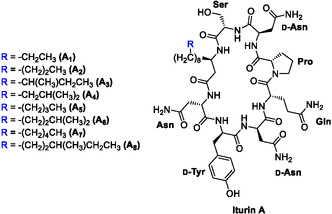
Structure of bacterially produced iturin A isoforms.

The most potent antifungal secondary metabolites produced by *Bacillus* spp. include the lipopeptides belonging to the iturin family, which consist of iturins, bacillomycins, mycosubtylin and mojavensin [5]. Among these, the iturin A family of compounds (Figure 1) is one of the most extensively studied since its first isolation from *B. subtilis* in 1950 [6] and its first structural characterisation in 1978 [7]. Iturin A exhibits strong antifungal activity against a plethora of fungal pathogens [8, 9, 10], along with potential antibacterial [11], antiviral [12], and anticancer [13] properties. Therefore, iturin A is considered a promising candidate for diverse applications across the health, agricultural, and food industry sectors [8].

Iturin A consists of seven α‐amino acids with a conserved stereochemical configuration and a β‐amino fatty acid, referred to as iturinic acid, featuring variable alkyl chain lengths (C_14_‐C_17_) [14]. The alkyl chain can be either linear or can exhibit terminal branching (iso or anteiso), resulting in eight structurally distinct isoforms, designated A_1_ through A_8_ (Figure 1) [8]. Its still elusive mechanism of action, is thought to involve membrane disruption through hydrogen bonding between the D‐Tyr residue and the fungal ergosterols, followed by pore formation by the β‐amino fatty acid side‐chain [15, 16]. Disruption of fungal growth has also been shown to result from the accumulation of reactive oxygen species and the induction of oxidative stress [9, 17]. Furthermore, recent molecular docking and molecular dynamics simulations have proposed β‐tubulin as a potential intracellular target of iturin A in fungi [18]. However, additional experimental validation is required to conclusively identify its molecular targets.

Structure–activity relationship studies have highlighted the essential roles of the D‐Tyr residue and of the iturinic acid in the lipopeptide's bioactivity. In fact, both *O*‐methylation of the D‐tyrosine residue [19] and substitution of iturinic acid with β‐alanine [20] individually result in a complete loss of bioactivity. The strictly conserved (*R*) configuration of the iturinic acid is also crucial for the antifungal activity, as we have recently proven that the (*S*) epimer is not bioactive against *Fusarium graminearum* and *Candida albicans* [21].

The incorporation of a trifluoromethyl moiety has become an increasingly valuable tool in peptide and protein engineering, providing unique physicochemical and biochemical properties [22, 23]. More specifically, the addition of a CF_3_ group can enhance the bioactivity [24, 25], hydrophobicity [26, 27], and metabolic stability [28] of the parent compound. However, this is not always the case, as there are reported instances of reduced bioactivity or metabolic stability following CF_3_ addition [28, 29]. Therefore, the impact of trifluoromethylation on peptides and proteins must be assessed on a case‐by‐case basis, as the influence of fluorine's strong electron‐withdrawing properties on neighbouring atoms and the effects of the conformational changes induced by the trifluoromethyl group remain challenging to predict [30]. Another significant advantage of incorporating a CF_3_ group is that it enables characterisation by ^19^F‐NMR, providing a powerful tool to investigate the peptide's mode of action [31]. A key advantage of ^19^F NMR in biological systems is its low background signal, since fluorine is virtually absent from living organisms [32]. Moreover, the ^19^F nucleus has a 100% natural abundance with sensitivity comparable to the ^1^H nucleus, a wide chemical shift range, and high sensitivity to the local chemical environment [32, 33].

**FIGURE 2:**
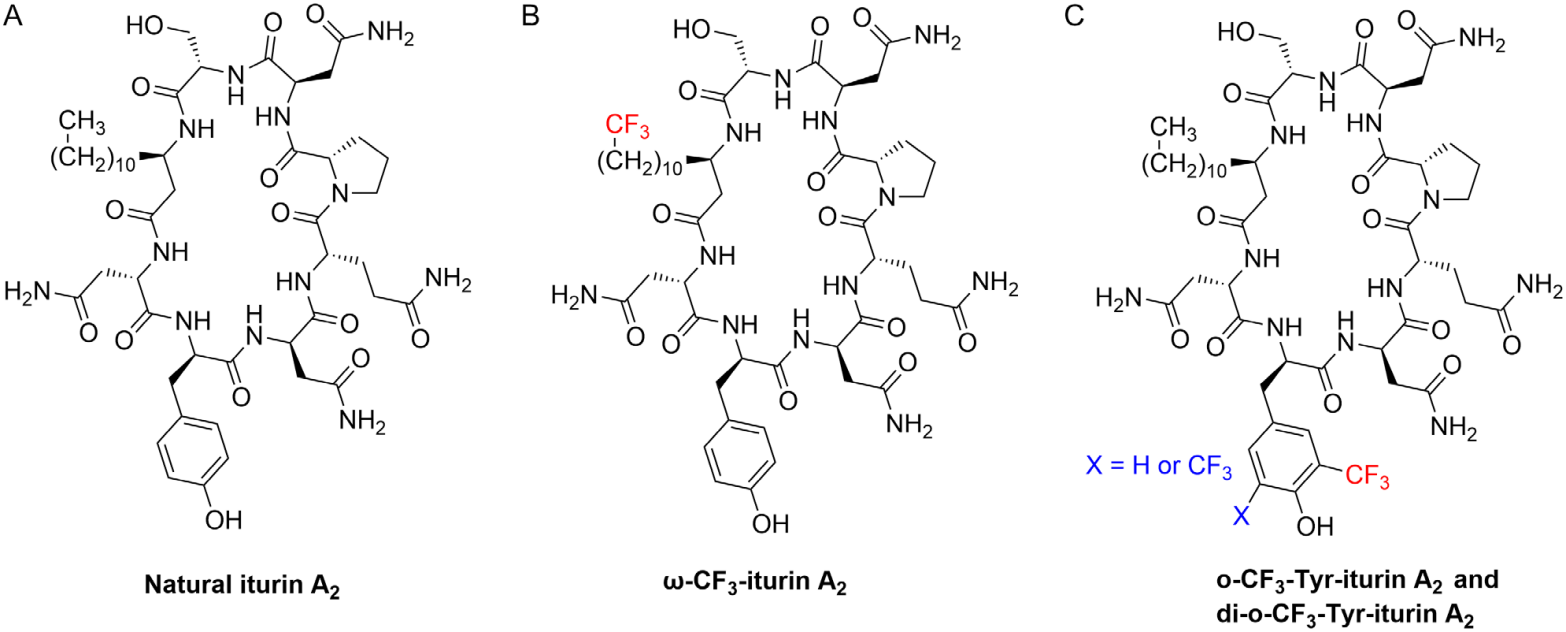
Structures of the lipopeptides synthesised in this work compared to natural iturin A_2_. (A) Natural iturin A_2_, (B) iturin A_2_ analogue with a terminal CF_3_ group in the alkyl side‐chain of iturinic acid, (C) iturin A_2_ with one or two CF_3_ groups in the *ortho* positions of the D‐Tyr residue.

In a previous study, we have developed an improved synthesis of iturin A_2_ (Figure 2A) that involves the asymmetric synthesis of the β‐amino fatty acid (iturinic acid), followed by solid‐phase peptide synthesis of the linear peptide and an on‐resin cyclisation reaction, with the use of orthogonal protecting groups [21]. In this work, we report the synthesis of three novel trifluoromethylated analogues of iturin A_2_ to explore the site‐specific impact of the trifluoromethylation on the antifungal activity of the lipopeptide and to provide novel ^19^F NMR spectroscopic probes to further investigate its mode of action. Trifluoromethylation was carried out on the residues considered most critical for bioactivity, namely iturinic acid (Figure 2B) and D‐tyrosine (Figure 2C) [19, 20].

## 2 Results and Discussion

### 2.1 Synthesis of the Trifluoromethylated Iturinic Acid

**SCHEME 1:**
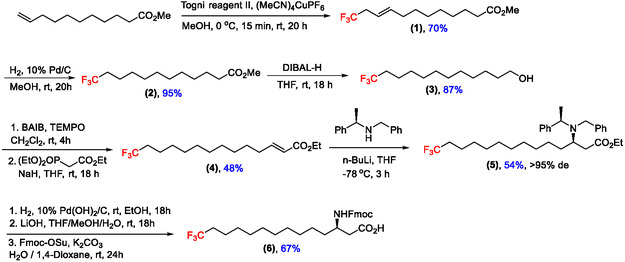
Enantioselective synthesis of Fmoc protected CF_3_‐iturinic acid.

Given the importance of iturinic acid for the bioactivity of iturin A, it was envisioned that its trifluoromethylation might improve several properties, such as the lipophilicity, metabolic stability, and membrane permeability of the lipopeptide [32]. Furthermore, it would provide a useful ^19^F NMR probe for mechanistic studies focused on this strictly conserved residue [33]. To this end, the asymmetric synthesis of a trifluoromethylated analogue of iturinic acid is described (Scheme 1), building on a previously established route to the parent compound [21].

The first step involved the Cu^I^‐catalysed allylic trifluoromethylation of commercial methyl undec‐10‐enoate [34]. This was achieved using Togni reagent II (1‐(trifluoromethyl)‐1,2‐benziodoxol‐3(1H)‐one), the hypervalent iodine species developed by Togni et al., as an electrophilic trifluoromethylating agent [35]. The resulting ester (**1**) was obtained in good yield (70%) and was then hydrogenated using standard methods [36], affording the saturated trifluoromethylated ester (**2**) in excellent yield (95%). This was followed by a reduction with diisobutylaluminum hydride (DIBAL‐H) to obtain the alcohol (**3**) in excellent yield (87%). Attempts to obtain the aldehyde selectively at –78°C were unsatisfactory and given the well‐documented propensity of DIBAL‐H to cause overreduction [37], full reduction to the alcohol was preferred. Selective oxidation to the corresponding aldehyde was performed with the use of (diacetoxyiodo)benzene (BAIB) as a stoichiometric oxidant and a catalytic amount of TEMPO [38]. The resulting aldehyde proved difficult to separate from the iodobenzene byproduct using flash column chromatography, and the crude mixture was therefore used in the next step without further purification. The α,β‐unsaturated ester (**4**) was synthesised via a Horner–Wadsworth–Emmons reaction [39], and the overall two‐step sequence afforded (**4**) in a yield of 48%.

The stereochemistry of the final product was effectively controlled through the addition of a homochiral lithium amide to (**4**), as developed by Davies et al. [40]. This process is known to afford excellent diastereoselectivities in related transformations [21, 40]. This lithium amide addition afforded (**5**) in a moderate yield (54%), after chromatographic purification. Analysis of the NMR spectra detected only one diastereomer and based on this and on previous results [21], it can be concluded that the required amino stereocentre has been installed with excellent stereocontrol (>95%). Finally, a hydrogenation, saponification and Fmoc protection sequence afforded the Fmoc protected trifluoromethylated (*R*)‐iturinic acid (**6**) in good yield (67%).

To the best of our knowledge, this is the first reported synthesis of a trifluoromethylated β‐amino fatty acid. Given that this residue is strictly conserved across all iturin‐type lipopeptides [5], it offers a powerful tool for modulating biochemical properties and for use as a ^19^F NMR probe across a broad class of related natural products.

### 2.2 Synthesis of Mono and Bis Trifluoromethylated D‐Tyrosine

Alongside iturinic acid, the D‐tyrosine residue also appears to play a pivotal role in the bioactivity of iturin A [19]. It was hypothesised that introducing a trifluoromethyl group at the ortho position of the D‐Tyr residue might give the aforementioned benefits of increased hydrophobicity, metabolic stability, and fluorine NMR compatibility to the lipopeptide [32]. Furthermore, the electron‐withdrawing CF_3_ group may enhance the hydrogen bond donating capacity of the adjacent hydroxyl group by exerting a strong inductive effect and by lowering its p*K*_a_ [41]. It is also important to note, that this substitution might impose a non‐negligible steric effect, as the trifluoromethyl group is considered comparable to an isopropyl moiety in size [41].

**SCHEME 2:**
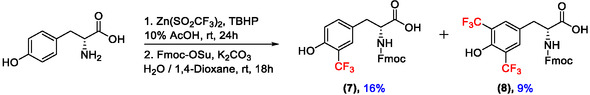
Synthesis of Fmoc protected trifluoromethylated D‐tyrosine.

Radical trifluoromethylation of unprotected D‐tyrosine was achieved using zinc trifluoromethanesulfinate (Baran's reagent) in the presence of tert‐butyl hydroperoxide as a stoichiometric oxidant [42]. This was followed by Fmoc protection of the resulting crude mixture (Scheme 2).

The obtained yields, although modest, are comparable with previous reports [43, 44]. Attempts to purify the trifluoromethylated products prior to the protection step resulted in decreased yields, therefore the crude mixture was used in the Fmoc protection reaction without prior purification and the final product was purified via HPLC (see Supporting Information). Attempts to use alternative conditions, specifically sodium trifluoromethanesulfinate (Langlois’ reagent) in combination with catalytic copper triflate [45], resulted in comparable overall yields, with a slight increase in selectivity toward the bis‐trifluoromethylated product, yielding 10% of compound (**7)** and 15% of compound (**8**). It is also worth noting that the radical trifluoromethylation could not be performed on the Fmoc protected amino acid, due to poor selectivity on the site of the aromatic trifluoromethylation that afforded complex mixtures (data not shown). While these results were deemed acceptable within the context of this study, future work should aim to further optimise the reaction in order to refine selectivity and enhance yields.

As bis‐trifluoromethylated tyrosine (**8**) was also formed in the reaction, it was deemed worthwhile to investigate the impact of two CF_3_ groups on the bioactivity of iturin A. In fact, the increase of the degree of fluorination, might also enhance the lipopeptide's suitability as a ^19^F NMR probe, particularly in complex biological environments, where stronger fluorine signals and the single ^19^F resonance arising from the product's symmetry are advantageous [31]. However, it cannot be excluded that the steric effects imposed by the presence of two CF_3_ moieties might alter the lipopeptide's conformation, potentially impacting its bioactivity.

### 2.3 Synthesis of Trifluoromethylated Iturin A_2_ Analogues

**SCHEME 3:**
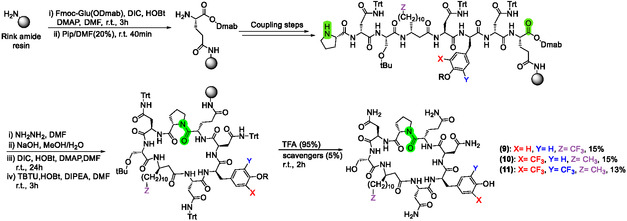
Solid‐phase peptide synthesis and on‐resin cyclisation of trifluoromethylated iturin A analogues. (**9)**: *ω*‐CF_3_ iturin A_2_, (**10**): *o‐*CF_3_‐Tyr iturin A_2_ (**11**): di‐*o‐*CF_3_‐Tyr iturin A_2_. For (**9**), R=tBu. For (**10**) and (**11**), R=H.

The synthesis of the trifluoromethylated lipopeptides was performed using our recently developed synthetic route to obtain iturin A_2_ analogues [21]. This route was inspired by the work of Gimenez et al [46]. on the synthesis of fengycin analogues and relies on the use of the Dmab protecting group, which is orthogonal to Fmoc solid‐phase peptide synthesis conditions and is selectively deprotected with a 5% hydrazine solution [47]. More specifically, it includes anchoring of the side chain of commercially available Fmoc‐Glu‐ODmab to a Rink amide resin, followed by Fmoc solid‐phase peptide synthesis of the linear peptide under standard conditions. The subsequent Dmab deprotection, on‐resin cyclisation and resin cleavage affords the final lipopeptide (Scheme 3). The cyclisation reaction was initially carried out using DIC/HOBt, as previously reported in the literature [46]. However, for the synthesis of iturin A_2_ analogues, a second cyclisation using TBTU/HOBt/DIPEA was required to drive the reaction to completion, as confirmed by LC‐MS analysis of the crude mixtures (data not shown). This additional step was likely required due to the presence of the sterically hindered proline residue at the N‐terminus.

**FIGURE 3:**
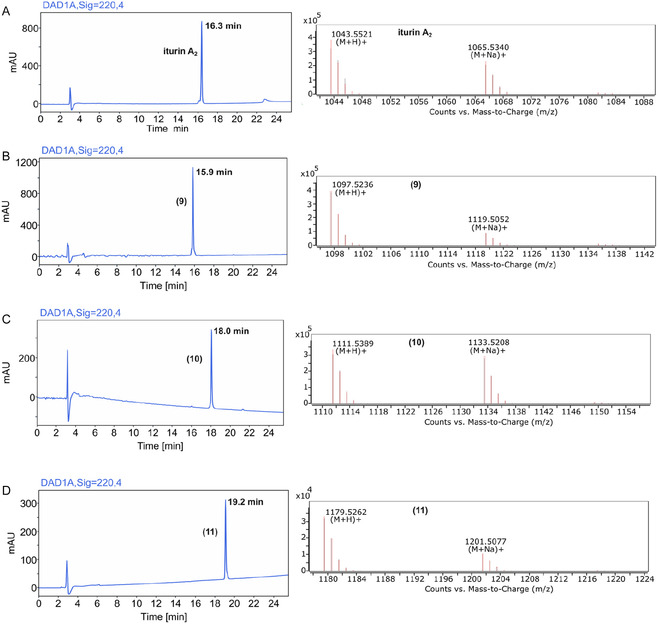
HPLC traces (left) and MS spectra (right) of the purified lipopeptides. (A) Natural iturin A_2_ (included for comparison), (B) (**9)**, *ω*‐CF_3_ iturin A_2_, (C) (**10**), *o‐*CF_3_‐Tyr iturin A_2_, (D) (**11**), di‐*o‐*CF_3_‐Tyr iturin A_2_.

The trifluoromethylated lipopeptides were obtained in good overall yields and excellent purity (≥95%), showcasing the robustness of this synthetic route for the synthesis of a wide range of iturinic lipopeptides (Figure 3).

HPLC retention times can be indicative of the relative hydrophobicity of the obtained trifluoromethylated lipopeptides, when compared to the parent compound [48]. It can be deduced that the substitution of a CH_3_ group with a CF_3_ moiety in iturin A's alkyl side‐chain did not result in an increase in hydrophobicity (Figure 3B). On the other hand, the substitution of an aromatic hydrogen with a CF_3_ group resulted in a pronounced increase in relative hydrophobicity (Figure 3C), which became even more pronounced upon introduction of two CF_3_ groups (Figure 3D).

**FIGURE 4:**
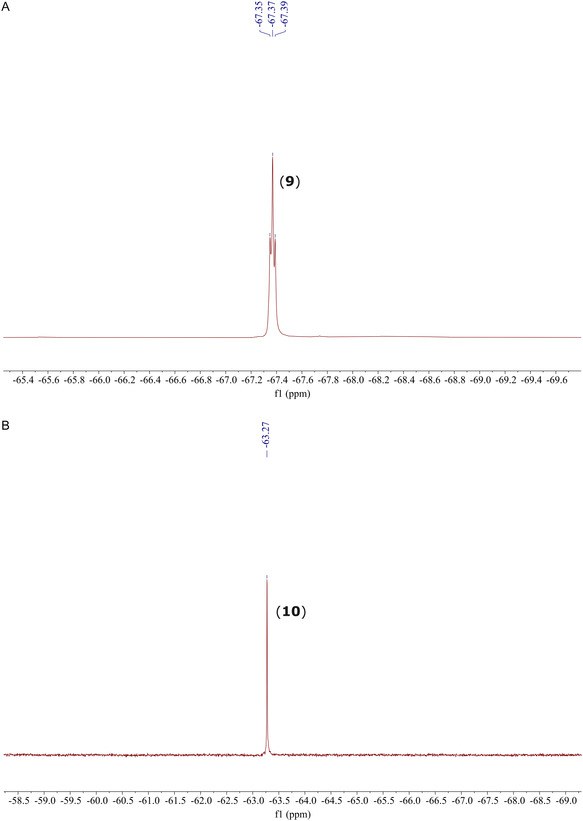
^19^F NMR spectra for (A) *ω*‐CF_3_ iturin A_2_ (**9**) and (B) *o*‐CF_3_ iturin A_2_ (**10**) in D_2_O containing 5% d_6_‐DMSO and Saboraud Dextrose Broth medium (30 g/L).

Lastly, ^19^F NMR spectra for lipopeptides (**9**) and (**10**) were obtained in D_2_O, containing Saboraud Dextrose Broth (Merck, 30 g/L), a medium commonly used for fungal cultivation (Figure 4) [49].

Compound (**9**) showed higher aqueous solubility (0.1 mM) than compound (**10**) (0.05 mM) and 5% d_6_‐DMSO was added to the samples to avoid precipitation. The well‐resolved, single signals obtained showcase the potential of these analogues for ^19^F NMR mechanistic studies in the context of biological systems.

### 2.4 Late‐Stage Trifluoromethylation of Iturin A

The semisynthesis of trifluoromethylated peptides via late‐stage modification represents an attractive strategy, enabling the rapid generation of fluorinated analogues for structure–activity relationship studies and compound library development. A recent comprehensive study by Ichiishi et al. showcased the application of the trifluoromethanesulfinate salts, under both oxidative and photocatalytic conditions, for the selective trifluoromethylation of aromatic residues in unprotected peptides [44].

**SCHEME 4:**
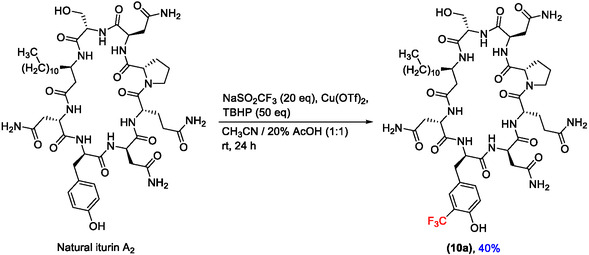
Late‐stage trifluoromethylation of iturin A_2_ with sodium trifluoromethanesulfinate and tert‐butyl hydro peroxide.

It was reasoned that iturin A would be an ideal target for this modification and that it could present an alternative to the total synthesis approach presented above. Initial attempts to perform this reaction with synthetically obtained iturin A_2_, using 5 equivalents of sodium trifluoromethanesulfinate and 10 equivalents of tert‐butyl hydro peroxide afforded only traces of the desired trifluoromethylated compound. Gratifyingly, when a large excess of reagents (50 eq) was used, the trifluoromethylated product was obtained in 40% isolated yield (Scheme 4).

The yield is considerably improved in comparison to previously reported semisyntheses of similar substrates conducted under oxidative conditions [44]. Moreover, comparison of the aromatic region of the ^1^H NMR spectra of the synthetic and the semisynthetic *o*‐CF_3_‐Tyr iturin A (**10**), coupled with LC‐MS analysis, revealed that the two compounds are structurally identical (Figure S1, Supporting Information).

The late‐stage trifluoromethylation reaction was also successfully performed on bacterially produced iturin A. A mixture of iturin A isoforms (Figure 1) containing an iturinic acid moiety with either 14 or 15 carbons, was isolated from a *Bacillus* sp. CS93 culture and was reacted as described above. LC‐MS analysis suggests a conversion of approximately 50%, comparable to the modification performed on the synthetic iturin A_2_ (Figure S2, Supporting Information). This late‐stage trifluoromethylation approach could also find application in the incorporation of isotopically labelled fluorine for bioimaging purposes.

### 2.5 Evaluation of Antifungal Activity

The antifungal activity of the obtained lipopeptides was evaluated by calculating the Minimum Inhibitory Concentration (MIC) against *Fusarium graminearum* and *Candida albicans*, compared to the natural lipopeptide (Table 1).

**TABLE 1:** MIC calculation for the three trifluoromethylated lipopeptides against *F. graminearum* and *C. albicans* compared to the natural lipopeptide. Amphotericin B was used as a positive control. Both commercially available iturin A (Merck) and synthetically obtained iturin A_2_ are included for reference. The assays were performed in triplicate.

Peptides	MIC. μM	
	*F. graminearum*	*C. albicans*
Synthetic iturin A_2_	16	64
Commercial iturin A	32	64
**(9)**: *ω*‐CF_3_ iturin A_2_	64	64
**(10)**: *o*‐CF_3_‐Tyr iturin A_2_	128	128
**(11)**: di‐*o*‐CF_3_‐Tyr iturin A_2_	>128	>128
Amphotericin B	4	2

*F. graminearum* is a filamentous fungus that causes the devastating cereal crop disease, known as Fusarium head blight [50]. It also produces mycotoxins that pose a threat to food safety and human health [51]. *Candida albicans* is a yeast responsible for approximately 70% of fungal infections worldwide and can sometimes lead to life‐threatening outcomes, particularly in high‐risk or immunocompromised patients [52]. The bioactivity of the obtained lipopeptides was compared to the synthetically obtained iturin A_2_, since the commercially available lipopeptide (Merck) contains a mixture of isoforms with different alkyl side‐chains (Figure 1) that might slightly influence bioactivity [21].

Interestingly, the *ω*‐CF_3_ iturin A_2_ analogue (**9**) displays a tenfold decrease in bioactivity against *F. graminearum*, while it completely retains the bioactivity of iturin A_2_ against *C. albicans*. The *o‐*CF_3_‐Tyr iturin A_2_ analogue (**10**) shows an tenfold decrease in bioactivity against *F. graminearum*, while only a slight twofold decrease against *C. albicans*. The di‐*o‐*CF_3_‐Tyr iturin A_2_ analogue (**11**) was not bioactive under the tested conditions. The pronounced increase in lipophilicity in (**10**) did not result in higher bioactivity, probably because of the “masking” of the essential hydroxyl moiety by the bulky trifluoromethyl group, combined with a possible change in the lipopeptide's conformation. Therefore, it might be unsurprising that the addition of a second trifluoromethyl group resulted in loss of bioactivity at least at the tested concentrations of up to 128 μM. Furthermore, it seems that the inhibition of *F. graminearum* by iturin A is more structure‐dependant than the inhibition of *C. albicans*, suggesting distinct mechanisms of action of the lipopeptide against these two microorganisms, possibly due to differences in the composition of the cell surfaces of the two fungi. Nevertheless, the obtained lipopeptides will undoubtedly serve as excellent fluorine NMR probes to gain insight into the mode of action of iturin A and future work should focus on performing these mechanistic studies.

## 3 Conclusions

In this study, three novel trifluoromethylated iturin A analogues were synthesised via site‐specific trifluoromethylation of D‐tyrosine and iturinic acid. The D‐tyrosine derivatives were obtained through a radical trifluoromethylation protocol, affording mono‐ and bis‐trifluoromethylated variants. The iturinic acid analogue was prepared via an electrophilic trifluoromethylation, followed by an asymmetric chiral auxiliary‐based route to the trifluoromethylated building block. This novel synthetic route for *ω*‐CF_3_ iturinic acid is anticipated to be applicable in the synthesis of analogues of other iturinic lipopeptides, as this moiety is strictly conserved across this compound family. Final cyclic lipopeptides were assembled using solid‐phase peptide synthesis and on‐resin cyclisation, with good yields and excellent purity. Additionally, a high‐yielding late‐stage aromatic trifluoromethylation was developed as an alternative route to this class of compounds.

HPLC analysis showed that the relative lipophilicity of the *o*‐CF_3_‐Tyr and di‐*o*‐CF_3_‐Tyr iturin A analogues increased proportionally to the increase in fluorine content, while the *ω*‐CF_3_ iturin A analogue showed a decrease in relative lipophilicity, when compared to the natural lipopeptide. Antifungal assays showed distinct activity profiles: o‐CF_3_‐Tyr analogues had reduced activity, especially against *F. graminearum*, while the *ω*‐CF_3_ analogue retained full activity against *C. albicans* despite diminished efficacy against *F. graminearum*. These findings might suggest organism‐specific modes of action for iturin A.

Lastly, the trifluoromethylated analogues obtained are expected to serve as valuable ^19^F NMR probes for elucidating the mechanism of action of this promising lipopeptide. The solid‐phase synthetic strategy employed for these lipopeptides is also expected to be broadly applicable to other iturinic lipopeptides and to the generation of libraries of iturin A analogues for structure–activity relationship studies.

## Supporting Information

Additional supporting information can be found online in the Supporting Information section. The authors have cited additional references within the Supporting Information [53, 54, 55, 56, 57, 58, 59]. **Supporting** **Fig.** **S1:** Comparison between the aromatic regions of the ^1^H NMR spectra (CD_3_OD, 400 MHz) of (A) trifluoromethylated iturin A (**10**) obtained through solid‐phase peptide synthesis and (B) trifluoromethylated iturin A (**10a**) obtained through late‐stage modification. **Supporting** **Fig.** **S2:** LC‐MS analysis (ESI‐TOF) of the late‐stage trifluoromethylation of bacterially produced iturin A.

## Conflicts of Interest

The authors declare no conflicts of interest.

## Supporting information

Supplementary Material

## Data Availability

The data that support the findings of this study are available in the supplementary material of this article.

## References

[1] M. C. Fisher , A. Alastruey‐Izquierdo , J. Berman , et al., “Tackling the Emerging Threat of Antifungal Resistance to Human Health,” Nature Reviews Microbiology 20 (2022): 557–571. 35352028 10.1038/s41579-022-00720-1 PMC8962932

[2] A. Vitiello , F. Ferrara , M. Boccellino , et al., “Antifungal Drug Resistance: An Emergent Health Threat,” Biomedicines 11 (2023): 1063. 37189681 10.3390/biomedicines11041063 PMC10135621

[3] F. Kaspar , P. Neubauer , and M. Gimpel , “Bioactive Secondary Metabolites from Bacillus Subtilis: A Comprehensive Review,” Journal of Natural Products 82 (2019): 2038–2053. 31287310 10.1021/acs.jnatprod.9b00110

[4] O. Lastochkina , M. Seifikalhor , S. Aliniaeifard , et al., “Bacillus Spp.: Efficient Biotic Strategy to Control Postharvest Diseases of Fruits and Vegetables,” Plants 8 (2019): 97. 31013814 10.3390/plants8040097 PMC6524353

[5] C. A. Dunlap , M. J. Bowman , and A. P. Rooney , “Iturinic Lipopeptide Diversity in the Bacillus Subtilis Species Group ‐ Important Antifungals for Plant Disease Biocontrol Applications,” Frontiers in Microbiology 10 (2019): 1794. 31440222 10.3389/fmicb.2019.01794 PMC6693446

[6] L. Delcambe , “Iturine, New Antibiotic Produced by Bacillus Subtilis,” Comptes Rendus des Seances de la Societe de Biologie et de ses Filiales 144 (1950): 1431–1434. 14812748

[7] F. Peypoux , M. Guinand , G. Michel , L. Delcambe , B. C. Das , and E. Lederer , “Structure of Iturine A, a Peptidolipid Antibiotic from Bacillus Subtilis,” Biochemistry 17 (1978): 3992–3996. 101232 10.1021/bi00612a018

[8] D. A. Yaraguppi , Z. K. Bagewadi , N. R. Patil , and N. Mantri , “Iturin: A Promising Cyclic Lipopeptide with Diverse Applications,” Biomolecules 13 (2023): 1515. 37892197 10.3390/biom13101515 PMC10604914

[9] S. Lei , H. Zhao , B. Pang , et al., “Capability of Iturin from Bacillus Subtilis to Inhibit Candida Albicans in Vitro and in Vivo,” Applied Microbiology and Biotechnology 103 (2019): 4377–4392. 30997554 10.1007/s00253-019-09805-z

[10] E. Arrebola , R. Jacobs , and L. Korsten , “Iturin A is the Principal Inhibitor in the Biocontrol Activity of Bacillus Amyloliquefaciens PPCB004 Against Postharvest Fungal Pathogens,” Journal of Applied Microbiology 108 (2010): 386–395. 19674188 10.1111/j.1365-2672.2009.04438.x

[11] K. T. R. and D. Sebastian , “Iturin and Surfactin from the Endophyte Bacillus Amyloliquefaciens Strain RKEA3 Exhibits Antagonism Against Staphylococcus Aureus,” Biocatalysis and Agricultural Biotechnology 36 (2021): 102125.

[12] E. V. Shekunov , P. D. Zlodeeva , S. S. Efimova , et al., “Cyclic Lipopeptides as Membrane Fusion Inhibitors Against SARS‐CoV‐2: New Tricks for Old Dogs,” Antiviral Research 212 (2023): 105575. 36868316 10.1016/j.antiviral.2023.105575 PMC9977712

[13] G. Dey , R. Bharti , G. Dhanarajan , et al., “Marine Lipopeptide Iturin A Inhibits Akt Mediated GSK3β and FoxO3a Signaling and Triggers Apoptosis in Breast Cancer,” Scientific Reports 5 (2015): 10316. 25974307 10.1038/srep10316 PMC4431395

[14] M. Ongena and P. Jacques , “ *Bacillus* lipopeptides: Versatile Weapons for Plant Disease Biocontrol,” Trends in Microbiology 16 (2008): 115–125. 18289856 10.1016/j.tim.2007.12.009

[15] L. Volpon , F. Besson , and J.‐M. Lancelin , “NMR Structure of Active and Inactive Forms of the Sterol‐Dependent Antifungal Antibiotic Bacillomycin L,” European Journal of Biochemistry 264 (1999): 200–210. 10447689 10.1046/j.1432-1327.1999.00605.x

[16] R. Maget‐Dana , M. Ptak , F. Peypoux , and G. Michel , “Pore‐forming Properties of Iturin A, a Lipopeptide Antibiotic,” Biochimica et Biophysica Acta (BBA) ‐ Biomembranes 815 (1985): 405–409. 3995034 10.1016/0005-2736(85)90367-0

[17] M. Hua , Q. Deng , M. Qiu , et al., “Iturin A Strongly Inhibits the Growth and T‐2 Toxin Synthesis of Fusarium oxysporum: A Morphological, Cellular, and Transcriptomics Study,” Foods 12 (2023): 1278. 36981204 10.3390/foods12061278 PMC10048737

[18] N. N. Cob‐Calan , L. A. Chi‐Uluac , F. Ortiz‐Chi , et al., “Molecular Docking and Dynamics Simulation of Protein β‐Tubulin and Antifungal Cyclic Lipopeptides,” Molecules 24 (2019): 3387. 31540347 10.3390/molecules24183387 PMC6767525

[19] R. Maget‐Dana , M. Ptak , F. Peypoux , and G. Michel , “Effect of the O‐methylation of the Tyrosine on the Pore‐Forming Properties of Iturins,” Biochimica et Biophysica Acta 898 (1987): 1–5. 3828330 10.1016/0005-2736(87)90104-0

[20] J. M. Bland , “The First Synthesis of a Member of the Iturin Family, the Antifungal Cyclic Lipopeptide, Iturin‐A2,” The Journal of Organic Chemistry 61 (1996): 5663–5664.

[21] P. Karamanis , M. Kiernan , J. Muldoon , et al., “Novel Synthesis of the Antifungal Cyclic Lipopeptide Iturin A and Its Fluorinated Analog for Structure‐Activity Relationship Studies,” Chemistry – A European Journal 31 (2025): e01341. 40711366 10.1002/chem.202501341 PMC12376246

[22] C. Jäckel and B. Koksch , “Fluorine in Peptide Design and Protein Engineering,” European Journal of Organic Chemistry 2005 (2005): 4483–4503.

[23] A. A. Berger , J. S. Völler , N. Budisa , and B. Koksch , “Deciphering the Fluorine Code‐The Many Hats Fluorine Wears in a Protein Environment,” Accounts of Chemical Research 50 (2017): 2093–2103. 28803466 10.1021/acs.accounts.7b00226

[24] F. Terzani , S. Belhattab , A. Le Guern , et al., “Synthesis and Biological Evaluation of Selective Pepstatin Based Trifluoromethylated Inhibitors of Cathepsin D,” European Journal of Medicinal Chemistry 267 (2024): 116178. 38295686 10.1016/j.ejmech.2024.116178

[25] I. Ojima , K. Kato , F. A. Jameison , et al., “New Potent Enkephalin Analogs Containing Trifluoromethylamino Acid Residues,” Bioorganic & Medicinal Chemistry Letters 2 (1992): 219–222.

[26] M. Oliver , C. Gadais , J. García‐Pindado , et al., “Trifluoromethylated Proline Analogues as Efficient Tools to Enhance the Hydrophobicity and to Promote Passive Diffusion Transport of the L‐Prolyl‐L‐Leucyl‐Glycinamide (PLG) tripeptide,” RSC Advances 8 (2018): 14597–14602. 35540789 10.1039/c8ra02511h PMC9079923

[27] C. Gadais , E. Devillers , V. Gasparik , E. Chelain , J. Pytkowicz , and T. Brigaud , “Probing the Outstanding Local Hydrophobicity Increases in Peptide Sequences Induced by Incorporation of Trifluoromethylated Amino Acids,” Chembiochem 19 (2018): 1026–1030. 29513394 10.1002/cbic.201800088

[28] V. Asante , J. Mortier , G. Wolber , and B. Koksch , “Impact of Fluorination on Proteolytic Stability of Peptides: a Case Study With α‐Chymotrypsin and Pepsin,” Amino Acids 46 (2014): 2733–2744. 25193166 10.1007/s00726-014-1819-7

[29] C. Pesenti , A. Arnone , S. Bellosta , et al., “Total Synthesis of a Pepstatin Analog Incorporating Two Trifluoromethyl Hydroxymethylene Isosteres (Tfm‐GABOB) and Evaluation of Tfm‐GABOB Containing Peptides as Inhibitors of HIV‐1 Protease and MMP‐9,” Tetrahedron 57 (2001): 6511–6522.

[30] J. M. Monkovic , H. Gibson , J. W. Sun , and J. K. Montclare , “Fluorinated Protein and Peptide Materials for Biomedical Applications,” Pharmaceuticals 15 (2022): 1201. 36297312 10.3390/ph15101201 PMC9609677

[31] D. Gimenez , A. Phelan , C. D. Murphy , and S. L. Cobb , “(19)F NMR as a Tool in Chemical Biology,” Beilstein Journal of Organic Chemistry 17 (2021): 293–318. 33564338 10.3762/bjoc.17.28 PMC7849273

[32] G. S. M. Hanson and C. R. Coxon , “Fluorinated Tags to Study Protein Conformation and Interactions Using 19F NMR,” Chembiochem 25 (2024): e202400195. 38744671 10.1002/cbic.202400195

[33] S. L. Cobb and C. D. Murphy , “19F NMR Applications in Chemical Biology,” Journal of Fluorine Chemistry 130 (2009): 132–143.

[34] A. T. Parsons and S. L. Buchwald , “Copper‐Catalyzed Trifluoromethylation of Unactivated Olefins,” Angewandte Chemie International Edition 50 (2011): 9120–9123. 21919144 10.1002/anie.201104053 PMC3390945

[35] J. Charpentier , N. Früh , and A. Togni , “Electrophilic Trifluoromethylation by Use of Hypervalent Iodine Reagents,” Chemical Reviews 115 (2015): 650–682. 25152082 10.1021/cr500223h

[36] C.‐H. Chiang , R. Ramu , Y.‐J. Tu , et al., “Regioselective Hydroxylation of C12‐C15 Fatty Acids with Fluorinated Substituents by Cytochrome P450 BM3,” Chemistry – A European Journal 19 (2013): 13680–13691. 24092541 10.1002/chem.201302402

[37] N. Z. Burns , P. S. Baran , and R. W. Hoffmann , “Redox Economy in Organic Synthesis,” Angewandte Chemie International Edition 48 (2009): 2854–2867. 19294720 10.1002/anie.200806086

[38] A. De Mico , R. Margarita , L. Parlanti , A. Vescovi , and G. Piancatelli , “A Versatile and Highly Selective Hypervalent Iodine (III)/2,2,6,6‐Tetramethyl‐1‐Piperidinyloxyl‐Mediated Oxidation of Alcohols to Carbonyl Compounds,” The Journal of Organic Chemistry 62 (1997): 6974–6977.

[39] D. Roman , M. Sauer , and C. Beemelmanns ,“Applications of the Horner‐Wadsworth‐Emmons Olefination in Modern Natural Product Synthesis,” Synthesis 53 (2021): 2713–2739.

[40] S. G. Davies , A. D. Smith , and P. D. Price , “The Conjugate Addition of Enantiomerically Pure Lithium Amides as Homochiral Ammonia Equivalents: Scope, Limitations, and Synthetic Applications,” Tetrahedron: Asymmetry 16 (2005): 2833–2891.

[41] B. E. Smart , “Fluorine Substituent Effects (on Bioactivity),” Journal of Fluorine Chemistry 109 (2001): 3–11.

[42] Y. Fujiwara , J. A. Dixon , F. O’Hara , et al., “Practical and Innate Carbon‐Hydrogen Functionalization of Heterocycles,” Nature 492 (2012): 95–99. 23201691 10.1038/nature11680 PMC3518649

[43] C. W. Kee , O . Tack , F. Guibbal , et al., “18F‐Trifluoromethanesulfinate Enables Direct C‐H 18F‐Trifluoromethylation of Native Aromatic Residues in Peptides,” Journal of the American Chemical Society 142 (2020): 1180–1185. 31913613 10.1021/jacs.9b11709 PMC6978814

[44] N. Ichiishi , J. P. Caldwell , M. Lin , et al., “Protecting Group Free Radical C‐H Trifluoromethylation of Peptides,” Chemical Science 9 (2018): 4168–4175. 29780547 10.1039/c8sc00368h PMC5941281

[45] B. R. Langlois , E. Laurent , and N. Roidot , “Trifluoromethylation of Aromatic Compounds with Sodium Trifluoromethanesulfinate Under Oxidative Conditions,” Tetrahedron Letters 32 (1991): 7525–7528.

[46] D. Gimenez , A. Phelan , C. D. Murphy , and S. L. Cobb , “Fengycin A Analogues with Enhanced Chemical Stability and Antifungal Properties,” Organic Letters 23 (2021): 4672–4676. 34077216 10.1021/acs.orglett.1c01387 PMC8289291

[47] W. C. Chan , B. W. Bycroft , D. J. Evans , and P. D. White , “A Novel 4‐Aminobenzyl Ester‐Based Carboxy‐Protecting Group for Synthesis of Atypical Peptides by Fmoc‐But Solid‐Phase Chemistry,” Journal of the Chemical Society, Chemical Communications 21 (1995): 2209–2210.

[48] J. Gregorc , N. Lensen , G. Chaume , J. Iskra , and T. Brigaud , “Trifluoromethylthiolation of Tryptophan and Tyrosine Derivatives: A Tool for Enhancing the Local Hydrophobicity of Peptides,” The Journal of Organic Chemistry 88 (2023): 13169–13177. 37672679 10.1021/acs.joc.3c01373 PMC10507666

[49] S. Basu , C. Bose , N. Ojha , et al., “Evolution of Bacterial and Fungal Growth Media,” Bioinformation 11 (2015): 182–184. 26124557 10.6026/97320630011182 PMC4479053

[50] L. E. Osborne and J. M. Stein , “Epidemiology of Fusarium Head Blight on Small‐Grain Cereals,” International Journal of Food Microbiology 119 (2007): 103–108. 17716761 10.1016/j.ijfoodmicro.2007.07.032

[51] Y. Chen , H. C. Kistler , and Z. Ma , “Fusarium Graminearum Trichothecene Mycotoxins: Biosynthesis, Regulation, and Management,” Annual Review of Phytopathology 57 (2019): 15–39. 10.1146/annurev-phyto-082718-100318 30893009

[52] J. Talapko , M. Juzbašić , T. Matijević , et al., “Candida Albicans‐The Virulence Factors and Clinical Manifestations of Infection,” Journal of fungi 7 (2021): 79. 33499276 10.3390/jof7020079 PMC7912069

[53] W. Zhang , Z. Zou , Y. Wang , et al., “Leaving Group Assisted Strategy for Photoinduced Fluoroalkylations Using N‐Hydroxybenzimidoyl Chloride Esters,” Angewandte Chemie International Edition 58 (2019): 624–627. 30444559 10.1002/anie.201812192

[54] S. Ozturk , C. C. Forneris , A. K. L. Nguy , E. J. Sorensen , and M. R. Seyedsayamdost , “Modulating OxyB‐Catalyzed Cross‐Coupling Reactions in Vancomycin Biosynthesis by Incorporation of Diverse D‐Tyr Analogues,” The Journal of Organic Chemistry 83 (2018): 7309–7317. 29806454 10.1021/acs.joc.8b00916

[55] E. Kaiser , R. L. Colescott , C. D. Bossinger , and P. I. Cook , “Color Test for Detection of Free Terminal Amino Groups in the Solid‐Phase Synthesis of Peptides,” Analytical Biochemistry 34 (1970): 595–598. 5443684 10.1016/0003-2697(70)90146-6

[56] P. Karamanis , J. Muldoon , C. D. Murphy , and M. Rubini , “Total Synthesis of Antifungal Lipopeptide Iturin A Analogues and Evaluation of their Bioactivity Against F. Graminearum,” Journal of Peptide Science 30 (2024): e3569. 38301277 10.1002/psc.3569

[57] N. K. O’Connor , A. S. Hudson , S. L. Cobb , et al., “Novel Fluorinated Lipopeptides from Bacillus sp. CS93 Via Precursor‐Directed Biosynthesis,” Amino Acids 46 (2014): 2745–2752. 25193167 10.1007/s00726-014-1830-z

[58] Clinical and Laboratory Standards Institute , Reference Method for Broth Dilution Antifungal Susceptibility Testing of Yeasts; Approved Standard, 2nd ed. (CLSI document M27‐A2, 2002).

[59] Clinical and Laboratory Standards Institute , Reference Method for Broth Dilution Antifungal Susceptibility Testing of Filamentous Fungi; Approved Standard, 1st ed. (CLSI document M38‐A, 2002).

