# Supplementary material for: Synthesis of Trifluoromethylated Analogues of the Cyclic Lipopeptide Iturin A and Evaluation of their Antifungal Activity
Source: Chempluschem. 2025 Dec 12;91(1):e202500590. doi: 10.1002/cplu.202500590 (PMC12777510; doi:10.1002/cplu.202500590)
Supplement: Supplementary file 1 — Supplementary Material [file CPLU-91-e202500590-s001.pdf]

Supporting Information

Synthesis of Trifluoromethylated Analogues of the Cyclic Lipopeptide Iturin A and Evaluation of their Antifungal Activity

Periklis Karamanis[a],[b], Matthew Kiernan[a],[b], Jimmy Muldoon[a], Paul Evans[a],[b], Cormac D. Murphy*[b],[c],[d], and Marina Rubini*[a],[b]

[a] P. Karamanis, M. Kiernan, J. Muldoon, P. Evans, M. Rubini

UCD School of Chemistry, University College Dublin, Dublin, Ireland

E-mail: marina.rubini@ucd.ie

[b] P. Karamanis, M. Kiernan, P. Evans, C.D. Murphy, M. Rubini

BiOrbic Bioeconomy SFI Research Centre, University College Dublin, Dublin, Ireland

[c] Cormac D. Murphy

UCD School of Biomolecular and Biomedical Science, University College Dublin, Dublin, Ireland

E-mail: cormac.d.murphy@ucd.ie

[d] Cormac D. Murphy

Conway Institute for Biomolecular and Biomedical Research, University College Dublin, Dublin, Ireland

Contents

General Methods....3

Synthesis of Fmoc protected CF_3-iturinic acid (**6**): 4

Methyl (*E*)-12,12,12-trifluorododec-9-enoate (**1**)....4

Methyl 12,12,12-trifluorododecanoate (**2**)....4

12,12,12-Trifluorododecan-1-ol (**3**)....5

Ethyl (*E*)-14,14,14-trifluorotetradec-2-enoate (**4**) 5

Ethyl (*R*)-3-(benzyl((*R*)-1-phenylethyl)amino)-14,14,14-trifluorotetradecanoate (**5**)....7

(*R*)-3-((((9*H*-fluoren-9-yl)methoxy)carbonyl)amino)-14,14,14-trifluorotetradecanoic acid (**6**)....8

Synthesis of Fmoc protected CF_3-D-Tyrosines (**7**) and (**8**)....9

Solid-phase peptide synthesis and cyclisation for trifluoromethylated cyclic lipopeptides (**9**), (**10**), and (**11**)....11

Late-stage Trifluoromethylation of Iturin A: 13

Isolation of Bacterially Produced Iturin A:....15

Late-stage Trifluoromethylation of Bacterially Produced Iturin A:....15

Antifungal Susceptibility Testing....16

NMR Spectra 18

References....31

General Methods

All protected amino acids (95% purity or higher) were purchased from Iris Biotech GMBH (Marktredwitz, Germany). Solvents and reagents (reagent grade or better) were purchased from Merck KGaA (Darmstadt, Germany). Chemical reactions were monitored using analytical thin-layer chromatography, performed using aluminium-backed silica plates (60 F254) and the stated eluents. Visualization was accomplished using a potassium permanganate stain. Product purification by flash column chromatography was performed using silica gel (Davisil, 230–400 mesh, 40–63 μm).

^{1}H NMR spectra were recorded using a Varian VnmrS (400 MHz) or a JEOL (400 MHz) spectrometer. Samples were dissolved in $CDCl_3$ or CD_3OD or d_6-DMSO and referenced to TMS (0.00 ppm). ^{13}C NMR spectra were recorded using a Varian VnmrS (101 MHz) or a JEOL (101 MHz) spectrometer. Samples were dissolved in $CDCl_3$ or CD_3OD or d_6-DMSO and referenced to TMS (0.00 ppm) or to the respective residual solvent peak. ^{19}F NMR spectra were recorded using a Varian VnmrS (376 MHz) or a JEOL (376 MHz) spectrometer. Samples were dissolved in $CDCl_3$ or CD_3OD or d_6-DMSO. Spectra were analysed using MestreNova 14.0. Chemical shifts are reported in parts per million (ppm) and coupling constants (*J*) are given in Hertz. Multiplicities are abbreviated as s (singlet), d (doublet), t (triplet), q (quartet), m (multiplet) or combinations thereof.

LC–MS data were acquired in ESI+ mode on an Agilent 6546 QToF system coupled with an Agilent 1260 Infinity Prime II LC system. Chromatography was carried out with a C_{18} column (Agilent Zorbax Eclipse Plus, 2.1 × 50 mm) using a binary solvent system with a linear gradient of acetonitrile and water (containing 0.1% formic acid) changing over 5 min from 10%–90% acetonitrile, followed by a final isocratic hold for 5 min. The flow rate was set at 0.6 mL/min. Data were processed with the Agilent Masshunter software. Target compounds were searched via compound matching using the Agilent FBF (Find-By Formula) algorithm, matching for singly charged monomeric ion species for common ions such as $[M+H]^+$ and $[M+Na]^+$.

Synthesis of Fmoc protected CF_3-iturinic acid (6):

Methyl (*E*)-12,12,12-trifluorododec-9-enoate (1)

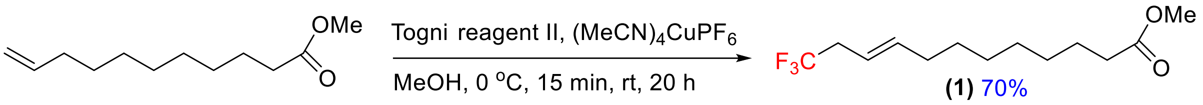

Togni reagent II (60% w/w, 938 mg, 1.78 mmol, 2 eq) and $(MeCN)_4CuPF_6$ (82 mg, 0.22 mmol, 0.25 eq) were added in a flame-dried round bottom flask under a N_2 atmosphere and were dissolved in anhydrous methanol (10 mL) The mixture was cooled to 0 °C and methyl-10-undecenoate (0.2 mL, 0.89 mmol, 1 eq) was added via syringe and the mixture colour changed to blue/green. The mixture was left stirring at room temperature for 20 hours. The solvent was removed under reduced pressure, and the crude product was dry-loaded on silica and purified via flash column chromatography (19:1 cyclohexane/ethyl acetate) to afford methyl (*E*)-12,12,12,-trifluorododec-9-enoate **(1)** as a colourless oil (166 mg, 0.62 mmol, 70%). $\mathbf{R_f}$ = 0.5 (19:1 cyclohexane/ethyl acetate); ^{1}**H NMR** (400 MHz, $CDCl_3$): δ 5.71-5.63 (m, 1H), 5.39-5.31 (m, 1H), 3.66 (s, 3H), 2.80-2.69 (m, 2H), 2.29 (t, *J*= 7.5 Hz, 2H), 2.03 (q, *J*= 7.2 Hz, 2H), 1.63-1.56 (m, 2H), 1.40-1.24 (m, 8H) ppm; ^{13}**C NMR** (101 MHz, $CDCl_3$): 174.4 (C), 138.3 (CH), 126.0 (q, *J*= 275 Hz, C), 117.5 (q, *J*= 3.5 Hz, CH), 51.4 (CH_3), 37.3 (q, *J*= 29 Hz, CH_2), 34.1 (CH_2), 32.4 (CH_2), 29.0 (2xCH_2), 28.9 (CH_2), 28.7 (CH_2), 24.9 (CH_2) ppm; ^{19}**F NMR** (376 MHz, $CDCl_3$): δ -66.79 (t, *J*= 10 Hz, CF_3) ppm; **HRMS** (ESI-TOF) calculated for $C_{13}H_{22}F_3O_2^+$ $[M+H]^+$: 267.1566, found: 267.1567. Data in agreement with literature.[1]

Methyl 12,12,12-trifluorododecanoate (2)

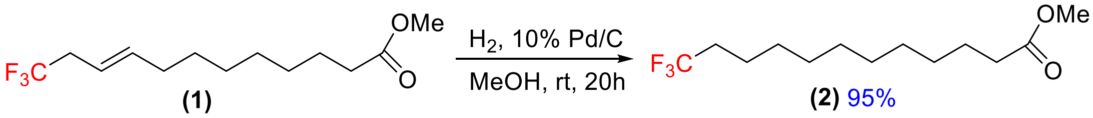

Under a hydrogen atmosphere (balloon), a solution of methyl (*E*)-12,12,12,-trifluorododec-9-enoate **(1)** (166 mg, 0.62 mmol, 1 eq) and 10% w/w palladium on carbon (130 mg, 0.12 mmol, 0.2 eq) in methanol (7 mL) was stirred at room temperature for 20 hours. The reaction mixture was filtered through celite with subsequent washes with methanol (3 x 10 mL), and the solvent was removed *in vacuo*. Methyl 12,12,12-trifluorododecanoate **(2)** was isolated as a colourless oil (158 mg, 0.59 mmol, 95%) and was used in the next step with no further purification. $\mathbf{R_f}$ = 0.5 (19:1 cyclohexane/ethyl acetate); ^{1}**H NMR** (400 MHz, $CDCl_3$): δ 3.66 (s, 3H), 2.29 (t, *J*=

7.4 Hz, 2H), 2.10-1.98 (m, 2H), 1.63-1.49 (m, 4H), 1.39-1.24 (m, 12H) ppm; **^{13}C NMR** (101 MHz, $CDCl_3$): 174.3 (C), 127.3 (q, *J*= 274 Hz, C), 51.4 (CH_3), 34.1 (CH_2), 33.7 (q, *J*= 28 Hz, CH_2), 29.3 (CH_2), 29.2 (CH_2), 29.1 (CH_2), 29.1 (CH_2), 29.0 (CH_2), 28.6 (CH_2), 24.9 (CH_2), 21.8 (q, *J*= 4 Hz, CH_2) ppm; **^{19}F NMR** (376 MHz, $CDCl_3$): δ -66.43 (t, *J*= 11 Hz, CF_3) ppm; **HRMS** (ESI-TOF) calculated for $C_{13}H_{24}F_3O_2^+$ $[M+H]^+$: 269.1723, found: 269.1725. Data in agreement with literature.[2]

12,12,12-Trifluorododecan-1-ol (3)

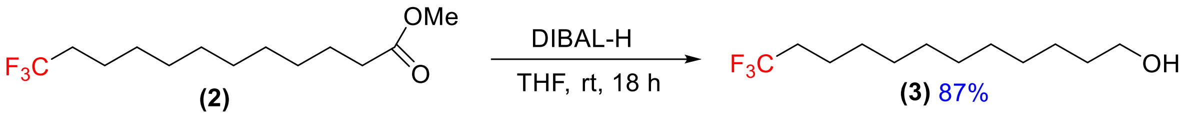

In a flame dried double-neck round bottom flask, methyl 12,12,12-trifluorododecanoate **(2)** (158 mg, 0.59 mmol, 1 eq) was dissolved in anhydrous tetrahydrofuran (7 mL) and cooled to 0 °C under nitrogen atmosphere. A diisobutylaluminum hydride solution (DIBAL-H, 1 M in hexanes, 1.2 mL, 1.20 mmol, 2 eq) was added dropwise via syringe and the mixture was left stirring at room temperature for 18 hours. The reaction was then quenched with MeOH (1 mL) and saturated sodium potassium tartrate solution (20 mL). The aqueous solution was then extracted with dichloromethane (3 x 20 mL). The combined organic layers were washed with brine, dried with Na_2SO_4 and the solvent was removed under reduced pressure. The resulting product 12,12,12-trifluorododecan-1-ol **(3)** was isolated as a white solid (123 mg, 0.51 mmol, 87%) and was used in the next steps with no further purification. **R_f** = 0.35 (9:1 cyclohexane/ethyl acetate); **^{1}H NMR** (400 MHz, $CDCl_3$): δ 3.63 (t, *J*= 6.7 Hz, 2H), 2.10-1.98 (m, 2H), 1.59-1.49 (m, 4H), 1.36-1.25 (m, 14H) ppm; **^{13}C NMR** (101 MHz, $CDCl_3$): δ 127.2 (q, *J*= 275 Hz, C), 63.1 (CH_2), 33.7 (q, *J*= 28 Hz, CH_2), 33.3 (CH_2), 29.5 (CH_2), 29.4 (CH_2), 29.4 (CH_2), 29.3 (CH_2), 29.1 (2xCH_2), 28.6 (CH_2), 21.8 (q, *J*= 3 Hz, CH_2) ppm; **^{19}F NMR** (376 MHz, $CDCl_3$): δ -66.45 (t, *J*= 11.3 Hz, CF_3) ppm. Data in agreement with literature.[3]

Ethyl (*E*)-14,14,14-trifluorotetradec-2-enoate (4)

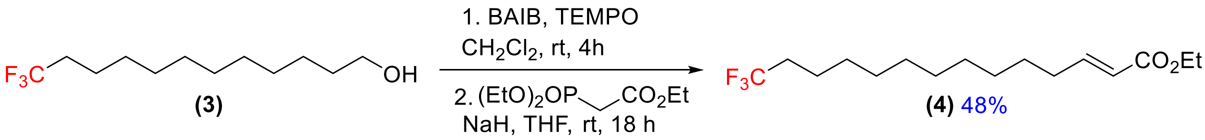

In a round bottom flask, 12,12,12-trifluorododecan-1-ol **(3)** (123 mg, 0.51 mmol, 1 eq) was dissolved in dichloromethane (10 mL) before (diacetoxyiodo)benzene (BAIB, 181 mg, 0.56 mmol, 1.1 eq) and 2,2,6,6-tetramethyl-1-piperidinyloxy (TEMPO, 8 mg, 0.05 mmol, 0.1 eq) were added to the mixture. The solution was left stirring at room temperature for 4 hours, before a further 0.1 eq (16 mg, 0.05 mmol) of BAIB and 0.1 eq of TEMPO (8 mg, 0.05 mmol) were added to the reaction. After stirring the reaction mixture at room temperature for 1 hour, TLC analysis (19:1 cyclohexane/ethyl acetate) showed complete consumption of the starting material and formation of a new product (**R_f** = 0.5). The reaction was then quenched by adding sodium sulfite (Na_2SO_3) solution (10 mL). The mixture was extracted with DCM (3 x 20 mL) and the combined organic layers were washed with brine (20 mL), dried with Na_2SO_4, filtered and concentrated *in vacuo*. The presence of the desired aldehyde was confirmed by ^{1}H NMR analysis of the crude product (data not shown).

In a separate flame dried double-neck round bottom flask and under anhydrous conditions at 0 ˚C, sodium hydride (90% w/w dispersion in oil, 34 mg, 1.28 mmol, 2.5 eq) was slowly added to a solution of triethyl phosphonoacetate (254 μL, 1.28 mmol, 2.5 eq) in anhydrous tetrahydrofuran (7 mL). The mixture was left stirring at 0 °C for 45 minutes. The crude yellow solid containing 12,12,12-trifluorododecanal, assuming 100% conversion in the previous step (122 mg, 0.51 mmol, 1 eq) was dissolved in anhydrous THF (2 mL) and was added dropwise to the mixture containing the phosphonate. The resulting mixture was allowed to warm up from 0 °C to room temperature and was left stirring for 18 hours. After quenching the reaction with water (10 mL), the aqueous layer was extracted with ethyl acetate (3 x 20 mL) and the combined organic layers were washed with brine (20 mL), dried over Na_2SO_4, filtered and concentrated *in vacuo*. The crude product was purified by flash chromatography (19:1 cyclohexane/ethyl acetate) to afford ethyl (*E*)-14,14,14-trifluorotetradec-2-enoate (**4**) as a colourless liquid (76 mg, 0.24 mmol, 48%). **R_f** = 0.6 (19:1 cyclohexane/ethyl acetate); **^{1}H NMR** (400 MHz, $CDCl_3$): δ 6.96 (dt, *J*= 15.7 Hz, 6.8 Hz, 1H), 5.80 (dt, *J*= 15.7 Hz, 2.0 Hz, 1H), 4.18 (q, *J*= 7.0 Hz, 2H), 2.22-2.16 (m, 2H), 2.12-1.99 (m, 2H), 1.57-1.51 (m, 2H), 1.47-1.41 (m, 2H), 1.34-1.26 (m, 15H) ppm; **^{13}C NMR** (101 MHz, $CDCl_3$): δ 166.8 (C), 149.4 (CH), 127.3 (q, *J*= 275 Hz, C), 121.2 (CH), 60.1 (CH_2), 33.7 (q, *J*= 28 Hz, CH_2), 32.2 (CH_2), 29.4 (CH_2), 29.3 (CH_2), 29.3 (CH_2), 29.1 (CH_2), 29.1 (CH_2), 28.7 (CH_2), 28.0 (CH_2), 21.8 (q, *J*= 2 Hz, CH_2), 14.3 (CH_3) ppm; **^{19}F NMR** (376 MHz, $CDCl_3$): δ -66.44 (t, *J*= 10.8 Hz, CF_3) ppm; **HRMS** (ESI-TOF) calculated for $C_{16}H_{28}F_3O_2^+$ [M+H]$^+$: 309.2036, found: 309.2038.

Ethyl (*R*)-3-(benzyl((*R*)-1-phenylethyl)amino)-14,14,14-trifluorotetradecanoate (5)

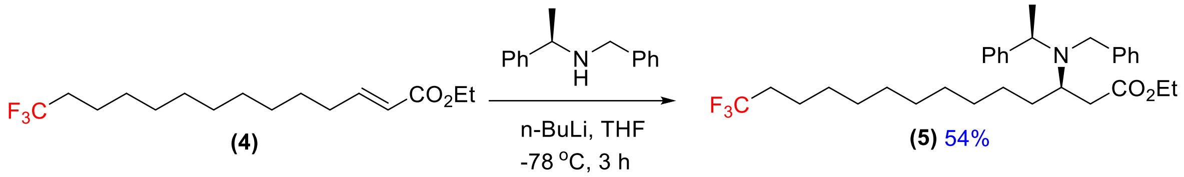

In a flame dried double-neck round bottom flask, under anhydrous conditions and at 0 °C, n-butyllithium (n-BuLi, 2.5 M in hexanes, 0.6 mL, 1.50 mmol) was added dropwise to a solution of (*R*)-*N*-benzyl-α-methylbenzylamine (0.3 mL, 1.44 mmol) in anhydrous THF (4 mL). The solution's colour changed to purple and was left stirring at 0 °C for 30 minutes. An aliquot of the resulting lithium amide solution (2.5 mL, 0.72 mmol, 3 eq) was added dropwise, under anhydrous conditions and at -78 °C to a solution of ethyl (*E*)-14,14,14-trifluorotetradec-2-enoate (**4**) (76 mg, 0.24 mmol, 1 eq) in anhydrous THF (4 mL). The resulting red-coloured mixture was left stirring at -78 °C for 4 hours. After quenching the reaction with saturated ammonium chloride solution (6 mL) and diluting with water (10 mL), the layers were separated. The mixture was extracted with diethyl ether (3 x 20 mL) and the combined organic layers were washed with HCl 1M (20 mL), saturated $NaHCO_3$ (20 mL), brine (20 mL), dried over Na_2SO_4, filtered and concentrated *in vacuo*. The crude product was purified by flash chromatography (19:1 cyclohexane: ethyl acetate) to afford ethyl (*R*)-3-(benzyl((*R*)-1-phenylethyl)amino)-14,14,14-trifluorotetradecanoate (**5**) (67 mg, 0.13 mmol, 54%) as a pale-yellow oil. $\mathbf{R_f}$ = 0.65 (19:1 cyclohexane/ethyl acetate); **^{1}H NMR** (400 MHz, $CDCl_3$): δ 7.42 (d, *J*= 7.2 Hz, 2H), 7.35-7.21 (m, 8H), 4.06-3.95 (m, 2H), 3.83 (q, *J*= 7.5 Hz, 1H), 3.78 (d, *J*= 15 Hz, 1H), 3.54 (d, *J*= 15 Hz, 1H), 3.29 (tt, *J*= 8.4 Hz, 4.2 Hz, 1H), 2.09-1.99 (m, 4H), 1.53-1.49 (m, 4H), 1.34-1.25 (m, 17H), 1.18 (t, *J*= 7.1 Hz, 3H) ppm; **^{13}C NMR** (101 MHz, $CDCl_3$): δ 172.9 (C), 143.2 (C), 141.8 (C), 128.2 (2 x CH), 128.2 (2 x CH), 128.0 (2 x CH), 127.9 (2 x CH), 126.9 (CH), 126.6 (CH), 60.1 (CH_2), 58.0 (CH), 54.1 (CH), 50.0 (CH_2), 36.8 (CH_2), 33.7 (q, *J*= 28 Hz, CH_2), 33.5 (CH_2), 29.6 (3 x CH_2), 29.5 (CH_2), 29.2 (CH_2), 28.7 (CH_2), 27.0 (CH_2), 21.8 (q, *J*= 3 Hz, CH_2), 19.7 (CH_3), 14.1 (CH_3) ppm; **^{19}F NMR** (376 MHz, $CDCl_3$): δ -66.42 (t, *J*= 10.8 Hz, CF_3) ppm; **HRMS** (ESI-TOF) calculated for $C_{31}H_{45}F_3NO_2^+$ $[M+H]^+$: 520.3397, found: 520.3399.

(*R*)-3-((((9*H*-fluoren-9-yl)methoxy)carbonyl)amino)-14,14,14-trifluorotetradecanoic acid (6)

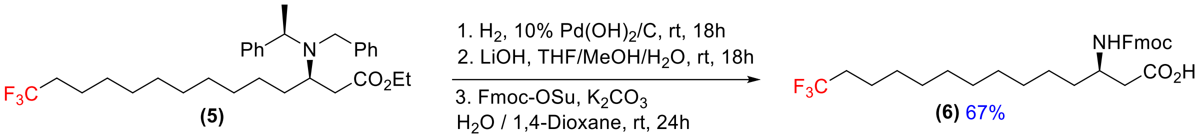

(5) **(6)** 67%

Under a hydrogen atmosphere (balloon), a solution of (*R*)-3-(benzyl((*R*)-1-phenylethyl)amino)-14,14,14-trifluorotetradecanoate (**5**) (67 mg, 0.13 mmol, 1 eq) and 20% w/w palladium hydroxide on carbon (21 mg, 0.03 mmol, 0.2 eq) in ethanol with 10% acetic acid (5 mL) was stirred at room temperature for 18 hours. The reaction mixture was filtered through celite with subsequent washes with ethanol (3 x 10 mL) and the solvent was removed *in vacuo*. TLC analysis (9:1 dichloromethane/methanol) confirmed the consumption of the starting material and the formation of a new product (R_f= 0.2).

The obtained crude pale-yellow oil was dissolved in THF (3 mL) and methanol (1 mL). An aqueous solution (3 mL) of lithium hydroxide (16 mg, 0.65 mmol, 5 eq) was slowly added to the mixture and it was left stirring at room temperature for 18 hours. The solvent was removed under reduced pressure and 10 mL of H_2O were added, followed by acidification with HCl (1 M) until pH=2. The resulting aqueous solution was extracted with ethyl acetate (3 x 20 mL) and the combined organic layers were washed with brine, dried under Na_2SO_4, filtered and concentrated *in vacuo*. TLC analysis (9:1 dichloromethane/methanol) confirmed the consumption of the starting material and the formation of a new product (R_f = 0.1 with streaking).

The obtained white solid was re-dissolved in 1:1 H_2O/1,4-dioxane (10 mL), potassium carbonate (54 mg, 0.39 mmol, 3 eq) was added (pH = 9) and the mixture was cooled to 0 °C. A solution of Fmoc-OSu (88 mg, 0.26 mmol, 2 eq) in 1,4-dioxane (3 mL) was added dropwise to the initial mixture at 0 °C and the resulting solution was allowed to warm up to room temperature and was kept stirring for 24 hours. 1,4-Dioxane was then removed under reduced pressure and the resulting aqueous solution was acidified with 1 M HCl until pH = 2. The solution was extracted with ethyl acetate (3 x 20 mL), washed with brine, dried under Na_2SO_4, filtered and concentrated *in vacuo*.

The crude solid was re-dissolved in MeOH and was purified via semi-preparative reversed-phase HPLC equipped with a C_{18} column (Agilent Zorbax SB-C_{18}, 9.4x250 mm) and a binary solvent system (solvent A: H_2O with 0.1% TFA, solvent B: Acetonitrile with 0.1% TFA). The

flow rate was set at 4 mL/min and UV detection was performed at 280 nm. The gradient started at 30% solvent B and increased linearly to 100% B over 20 minutes, followed by an isocratic hold at 100% B for 3 minutes. The solvent of the combined fractions was removed under reduced pressure to afford (*R*)-3-((((9*H*-fluoren-9-yl)methoxy)carbonyl)amino)-14,14,14-trifluorotetradecanoic acid (**6**) (45 mg, 0.087 mmol, 67%) as a white solid. **Retention time**: 16.1 min; **R_f** = 0.5 with streaking (9:1 dichloromethane/methanol); **^{1}H NMR** (500 MHz, $CDCl_3$): δ 7.75 (d, *J*= 7.5 Hz, 2H), 7.58 (d, *J*= 7.5 Hz, 2H), 7.39 (t, *J*= 7.3 Hz, 2H), 7.30 (t, *J*= 7.3 Hz, 2H), 5.13 (d, *J*= 8.8 Hz, 1H), 4.40 (d, *J*= 6.7 Hz, 2H), 4.22 (t, *J*= 6.7 Hz, 1H), 3.99-3.92 (m(br), 1H), 2.64-2.54 (m, 2H), 2.09-1.99 (m, 2H), 1.56-1.50 (m, 4H), 1.33-1.24 (m, 15H) ppm; **^{13}C NMR** (125 MHz, $CDCl_3$): δ 176.1 (C), 156.0 (C), 143.9 (2 x C), 141.3 (2 x C), 127.7 (2 x CH), 127.3 (q, *J*= 275 Hz, C), 126.2 (2 x CH), 125.0 (2 x CH), 120.0 (2 x CH), 66.7 (CH_2), 48.0 (CH), 47.3 (CH), 38.9 (CH_2), 34.4 (CH_2), 33.7 (q, *J*= 28 Hz, CH_2), 29.7 (CH_2), 29.4 (CH_2), 29.3 (CH_2), 29.2 (CH_2), 29.1 (CH_2), 28.7 (CH_2), 26.1 (CH_2), 21.8 (q, *J*= 3 Hz, CH_2) ppm; **^{19}F NMR** (470 MHz, $CDCl_3$): δ -66.41 (t, *J*= 10.4 Hz, CF_3) ppm; **HRMS** (ESI-TOF) calculated for $C_{29}H_{37}F_3NO_4^+$ $[M+H]^+$: 520.2669, found: 520.2673.

Synthesis of Fmoc protected CF_3-D-Tyrosines (7) and (8)

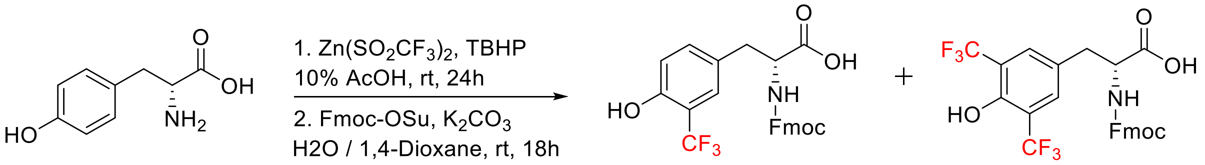

(7) 16% **(8)** 9%

Under nitrogen atmosphere, D-tyrosine (200 mg, 1.104 mmol, 1 eq) and zinc trifluoromethanesulfinate (1.1 g, 3.312 mmol, 3 eq) were suspended in degassed 10% aqueous AcOH solution (20 mL). *Tert*-Butyl hydroperoxide (TBHP, 70% in H_2O, 0.77 mL, 5.52 mmol, 5 eq) was added dropwise a degassed 10% aqueous AcOH solution (8.85 mL). The TBHP solution was slowly added to the initial cloudy mixture via a syringe pump (Fisherbrand KDS100 Legacy) at a rate of 0.8 mL/h under minimal stirring. After 18 h the solution had become clear, and it was added to two separate Waters Sep-Pak C18 cartridges activated with MeOH (2 mL) and H_2O (10 mL). The crude mixture was added to the cartridges, washed with H_2O (10 mL) and the products were eluted with MeOH (10 mL), followed by solvent removal under reduced pressure.

The resulting yellow solid was re-dissolved in H_2O (10 mL) and 1,4-dioxane (5 mL). Potassium carbonate (457 mg, 3.312 mmol, 3 eq) was added (pH = 9) and the mixture was cooled to 0 °C. Fmoc-OSu (745 mg, 2.208 mmol, 2 eq) was dissolved in 1,4-dioxane (10 mL) and was added dropwise to the mixture at 0 °C over 30 minutes. The mixture was left stirring at room temperature for 18 h and 1,4-dioxane was removed under reduced pressure. The aqueous solution was then extracted with cold Et_2O (3 x 20 mL), acidified with 1 M HCl (pH = 2) and extracted with EtOAc (3 x 30 mL). The combined organic layers were washed with brine (20 mL), dried under Na_2SO_4, filtered and the solvent was removed under reduced pressure to afford a yellow solid (300 mg).

The crude solid was re-dissolved in MeOH (10 mg/mL) and was purified via semi-preparative reversed-phase HPLC equipped with a C_{18} column (Agilent Zorbax SB-C_{18}, 9.4x250 mm) and a binary solvent system (solvent A: H_2O with 0.1% TFA, solvent B: Acetonitrile with 0.1% TFA). The flow rate was set at 4 mL/min and UV detection was performed at 280 nm. The gradient started at 30% solvent B and increased linearly to 100% B over 20 minutes, followed by an isocratic hold at 100% B for 3 minutes. The solvent of the combined fractions was removed under reduced pressure to afford products (**7**) (83 mg, 16%) and (**8**) (54 mg, 9%) as a pale yellow solid and a white solid respectively.

(7): Retention time = 12.3 min; **^{1}H NMR** (400 MHz, d_6-DMSO): δ 10.43 (s(br), 1H), 7.82 (d, *J*= 7.6 Hz, 2H), 7.68 (d, *J*= 8.5 Hz, 1H), 7.58 (t, *J*= 8 Hz, 2H), 7.38-7.33 (m, 3H), 7.30-7.21 (m, 3H) 6.88 (d, *J*= 8.3 Hz, 1H), 4.16-4.04 (m, 4H), 2.98 (dd, *J*= 14 Hz, 4.5 Hz, 1H), 2.76 (dd, *J*= 14 Hz, 10.2 Hz, 1H) ppm; **^{13}C NMR** (101 MHz, d_6-DMSO): δ 173.7 (C), 156.5 (C), 154.8 (C) 144.2 (C), 144.2 (C), 141.2 (C), 141.2 (C), 134.8 (CH), 128.6 (C), 128.2 (2xCH), 127.7 (CH), 125.8 (2xCH), 125.7 (2xCH), 124.6 (q, *J*= 271 Hz, C), 120.6 (2xCH), 117.3 (CH), 115.5 (q, *J*= 29 Hz, C), 66.2 (CH_2), 56.1 (CH), 47.1 (CH), 35.8 (CH_2); **^{19}F NMR** (376 MHz, d_6-DMSO): δ -63.26 (s, CF_3) ppm; **HRMS** (ESI-TOF) calculated for $C_{25}H_{20}F_3NO_5^+$ $[M+H]^+$: 472.1366, found: 472.1366. Data in agreement with literature.[4]

(8): Retention time = 13.9 min; **^{1}H NMR** (400 MHz, d_6-DMSO): δ 7.82 (d, *J*= 7.6 Hz, 2H), 7.79 (d, *J*= 8.5 Hz, 1H), 7.75 (s, 2H), 7.56 (t, *J*= 8 Hz, 2H), 7.35 (td, *J*= 7.4 Hz, 1.6 Hz, 2H), 7.26-7.18 (m, 2H), 4.18-4.08 (m, 4H), 3.10 (dd, *J*= 14 Hz, 4.2 Hz, 1H), 2.86 (dd, *J*= 14 Hz, 10.5 Hz, 1H) ppm; **^{13}C NMR** (101 MHz, d_6-DMSO): δ 173.4 (C), 156.6 (C), 152.1 (C), 144.2 (C), 144.1 (C), 141.2 (C), 141.2 (C), 132.1 (q, *J*= 5 Hz, 2 x CH), 130.7 (C), 128.2 (2 x CH), 127.6 (CH), 127.5 (CH), 125.7 (CH), 125.6 (CH), 123.9 (q, *J*= 271 Hz, 2 x C), 120.6 (2 x CH),

120.6 (q, *J*= 29 Hz, 2 x C), 66.3 (CH_2), 55.6 (CH), 47.0 (CH), 35.6 (CH_2) ppm; **^{19}F NMR** (376 MHz, d_6-DMSO): δ -63.05 (s, 2 x CF_3); **HRMS** (ESI-TOF) calculated for $C_{26}H_{21}F_6NO_5^+$ $[M+H]^+$: 540.1240, found: 540.1236.

Solid-phase peptide synthesis and cyclisation for trifluoromethylated cyclic lipopeptides (9), (10), and (11)

(**9**): The synthesis of the lipopeptides was performed similarly to a previously described method.[5] The Fmoc protected β-amino fatty acid (Fmoc-iturinic acid) was also obtained as previously described. Fmoc-Rink Amide resin (50 mg, 0.032 mmol, 0.64 mmol/g, 100-200 mesh, 1 eq) was swollen in DCM (2 mL) for 30 minutes and the Fmoc deprotection was performed with the addition of 20% piperidine in DMF (2 mL) with shaking for 40 minutes. The resin was washed with DMF and DCM (2 mL) and the deprotection step was repeated once. The resin was then washed three times with DMF and DCM (2 mL) and the solvent was filtered with a stream of N_2 gas. A solution of Fmoc-Glu-ODmab (100 mg, 0.160 mmol, 5 eq), DIC (30 µL, 0192 mmol, 6 eq), HOBt x H_2O (30 mg, 0.192 mmol, 6 eq) and DMAP (1 mg, 0.008 mmol, 0.25 eq) in DMF (2 mL) was added to the resin and the heterogeneous mixture was left shaking at 200 rpm for 18 hours at room temperature. The solvent was removed via filtration and the resin was washed three times with DMF and DCM (2 mL). This was followed by Fmoc deprotection with 20% piperidine in DMF (2 mL) and this step was performed twice as described above. The coupling and deprotection steps were repeated with the following amino acids: Fmoc-D-Asn(Trt)-OH (58 mg, 0.096 mmol, 3 eq), Fmoc-D-Tyr(tBu)-OH (44 mg, 0.096 mmol, 3 eq), Fmoc-Asn(Trt)-OH (58 mg, 0.096 mmol, 3 eq), Fmoc-CF_3-iturinic acid (**6**) (45 mg, 0.087 mmol, 2.7 eq), Fmoc-Ser(tBu)-OH (37 mg, 0.096 mmol, 3 eq), Fmoc-D-Asn(Trt)-OH (58 mg, 0.096 mmol, 3 eq), Fmoc-Pro-OH (33 mg, 0.096 mmol, 3 eq) in the presence of DIC (25 µL, 0.160 mmol, 5 eq), HOBt x H_2O (25 mg, 0.160 mmol, 5 eq) and DMAP (1 mg, 0.008 mmol, 0.25 eq). Each coupling was performed for 3 hours at room temperature, except for (**6**) which was left for 18 hours at room temperature. Coupling and deprotection were routinely monitored via the ninhydrin (Kaiser) test.[6]

After the final Fmoc deprotection the resin was treated with a 5% hydrazine solution in DMF (2 mL) for 5 minutes and the process was repeated four times. The resin was subsequently washed five times with DMF (2 mL). Then, the resin was incubated in a 5 mM NaOH solution

in H_2O/MeOH 1:1 (3 mL) to ensure complete elimination of the Dmab protecting group. The resin was washed three times with DMF and DCM (2 mL) and was reacted with DIC (15 µL, 0.096 mmol, 3 equiv.), HOBt x H_2O (15 mg, 0.096 mmol, 3 eq) and DMAP (1 mg, 0.008 mmol, 0.25 eq) in DMF (2 mL) at room temperature for 18 hours. The resin was then washed three times with DMF and DCM (2 mL) and another cyclisation step was performed with a solution of TBTU (31 mg, 0.096 mmol, 3 eq), HOBt x H_2O (15 mg, 0.096 mmol, 3 eq) and DIPEA (28 µL, 0.160 mmol, 5 eq) in DMF (2 mL) for 3 hours at room temperature. After the final three resin washes with DMF and DCM (2 mL) the resin was cleaved with a 95% TFA and 5% scavengers (TIPS, H_2O, phenol) solution (1 mL) and the cleavage step was performed twice. This was followed by filtration, concentration under N_2 gas, cold ether precipitation and centrifugation, affording the crude lipopeptide mixture. The mixture was re-dissolved in MeOH (5 mL) and was purified via HPLC equipped with a C_{18} column (Agilent Zorbax SB-C_{18}, 9.4x250 mm) using a binary solvent system (solvent A: H_2O with 0.1% TFA, solvent B: Acetonitrile with 0.1% TFA). The gradient started at 10% solvent B increasing linearly to 100% B over 40 minutes, followed by a final isocratic hold at 100% B for 5 min. The flow rate was set at 4 mL/min with UV detection at 220 nm. Lyophilisation of the purified fractions afforded lipopeptide (**9**) (5 mg, 4.6 µmol, yield 15%, purity ~95%) as a white solid. **Retention time**: 15.9 min; **^{19}F NMR** (376 MHz, CD_3OD): δ -67.64 (t, *J*= 11.4 Hz, CF_3) ppm; **HRMS** (ESI-TOF) calculated for $C_{48}H_{72}F_3N_{12}O_{14}^+$ $[M+H]^+$: 1097.5238, found: 1097.5236.

(10): The synthesis was performed as described above for (**9**), with the substitution of Fmoc-D-Tyr(tBu)-OH with Fmoc-trifluoromethyl tyrosine (**7**) (45 mg, 0.096 mmol, 3 eq) and Fmoc-CF_3-iturinic acid (**6**) with Fmoc-iturinic acid (45 mg, 0.096 mmol, 3 eq). All of the coupling steps were performed for 3 hours at room temperature. HPLC purification and lyophilisation of the collected fractions yielded the lipopeptide (**10**) (5.5 mg, 4.9 µmol, yield 15%, purity 96%) as a white solid. **Retention time**: 18.0 min; **^{19}F NMR** (376 MHz, CD_3OD): δ -63.61 (s, CF_3) ppm; **HRMS** (ESI-TOF) calculated for $C_{49}H_{74}F_3N_{12}O_{14}^+$ $[M+H]^+$: 1111.5394, found: 1111.5389.

(11): The synthesis was performed as described above for (**9**), with the substitution of Fmoc-D-Tyr(tBu)-OH with Fmoc-bis-trifluoromethyl tyrosine (**8**) (52 mg, 0.096 mmol, 3 eq) and Fmoc-CF_3-iturinic acid (**6**) with Fmoc-iturinic acid (45 mg, 0.096 mmol, 3 eq). All of the coupling steps were performed for 3 hours at room temperature. HPLC purification and lyophilisation of the collected fractions yielded the lipopeptide (**11**) (5 mg, 4.2 µmol, yield

13%, purity 98%) as a white solid. **Retention time**: 19.2 min; **HRMS** (ESI-TOF) calculated for $C_{50}H_{73}F_6N_{12}O_{14}^+$ $[M+H]^+$: 1179.5268, found: 1179.5262.

Late-stage Trifluoromethylation of Iturin A:

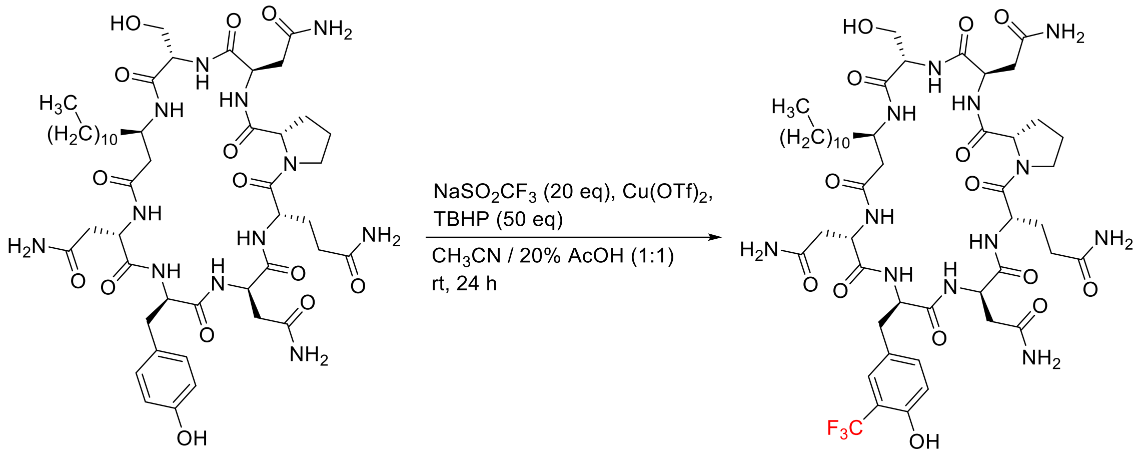

Natural iturin A_2 — **(10a)**, 40%

Iturin A_2 was obtained through solid-phase peptide synthesis and on-resin cyclisation using a previously described method and similarly to the method described above. Iturin A_2 (5 mg, 4.8 µmol, 1 eq), $NaSO_2CF_3$ (15 mg, 96 µmol, 20 eq) and $Cu(OTf)_2$ (1 mg, 2.8 µmol, 0.6 eq) were placed in a 2 mL Eppendorf tube and were dissolved in degassed 1:1 CH_3CN/ 20% AcOH (1 mL). A solution of Tert-butyl hydroperoxide (70% in H_2O, 33 µL, 0.24 mmol, 50 eq) in degassed 1:1 CH_3CN/ 20% AcOH (0.3 mL) was added dropwise to the initial mixture. The reaction was left for 24 hours at room temperature without stirring and it was added to a Waters Sep-Pak C18 cartridge activated with 2 mL MeOH and 10 mL H_2O. The crude mixture was added to the cartridge, washed with 10 mL of H_2O and the product was eluted with 10 mL MeOH. HPLC purification, using the method described for the synthetic lipopeptides, and lyophilisation afforded lipopeptide (**10a**) (2.1 mg, 1.9 µmol, yield 40%, purity 95%) as a white solid. **Retention time**: 18.0 min; **HRMS** (ESI-TOF) calculated for $C_{49}H_{74}F_3N_{12}O_{14}^+$ $[M+H]^+$: 1111.5394, found: 1111.5392. Characterisation data for the synthetic and the semi-synthetic trifluoromethylation reaction matched (**Fig. S1**).

(A)

(B)

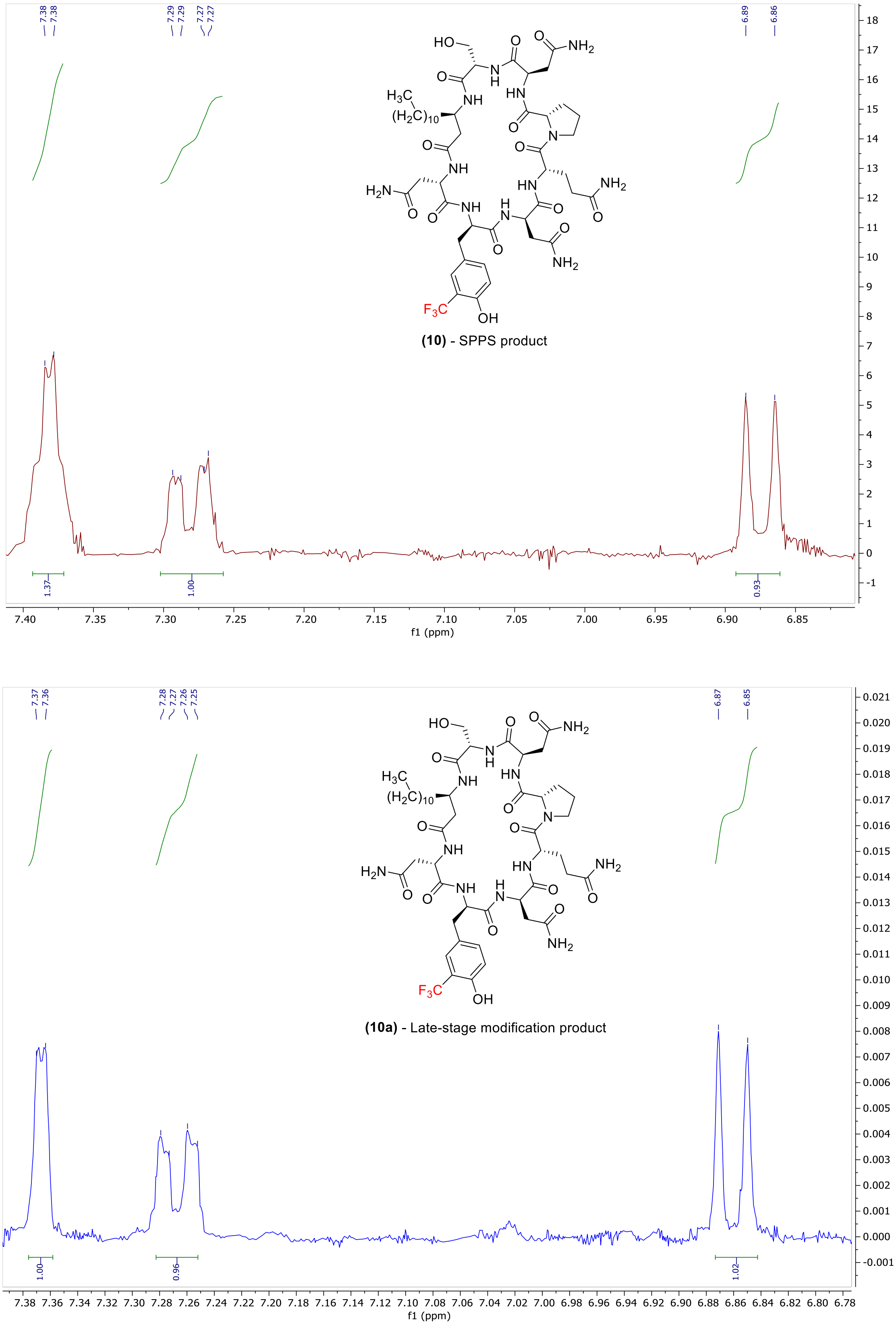

Figure S1: Comparison between the aromatic regions of the ^{1}H NMR spectra (CD_3OD, 400 MHz) of (A) trifluoromethylated iturin A (**10**) obtained through solid-phase peptide synthesis and (B) trifluoromethylated iturin A (**10a**) obtained through late-stage modification.

Isolation of Bacterially Produced Iturin A:

Bacterially produced iturin A (mixture of two isoforms) was isolated using a previously described method.[7] Briefly, *Bacillus* sp. CS93 was grown in 50 mL of Medium Optimised for Lipopeptide Production (MOLP)[8] for 72 hours at 30 °C. After centrifugation at 9500 rpm for 30 minutes at 4 °C, the supernatant was recovered and the lipopeptides were acid precipitated with a 5 M HCl solution (pH = 2). Then, the mixture was centrifuged and the precipitate was collected and re-suspended in 10 mL MeOH. The resulting mixture was filtered and the filtrate was concentrated under a stream of N_2 gas. The obtained crude lipopeptide mixture was dissolved in MeOH at a final concentration of 10 mg/mL. HPLC purification was conducted using a C_{18} column (Agilent Zorbax SB-C_{18}, 9.4x250 mm) using a binary solvent system (solvent A: H_2O with 0.1% TFA, solvent B: Acetonitrile with 0.1% TFA). The gradient started at 10% solvent B increasing linearly to 100% B over 70 minutes, followed by a final isocratic hold at 100% B for 5 min. The flow rate was set at 4 mL/min with UV detection at 220 nm. The fractions containing iturin A (retention time: 27-29 min) were collected and lyophilised, affording 5 mg of a mixture of C_{14}-iturin A and C_{15}-iturin A as a white solid.

C_{14}-iturin A: **HRMS** (ESI-TOF) calculated for $C_{48}H_{75}N_{12}O_{14}^+$ $[M+H]^+$: 1043.5520, found: 1043.5518.

C_{15}-iturin A: **HRMS** (ESI-TOF) calculated for $C_{49}H_{77}N_{12}O_{14}^+$ $[M+H]^+$: 1057.5677, found: 1057.5674.

Late-stage Trifluoromethylation of Bacterially Produced Iturin A:

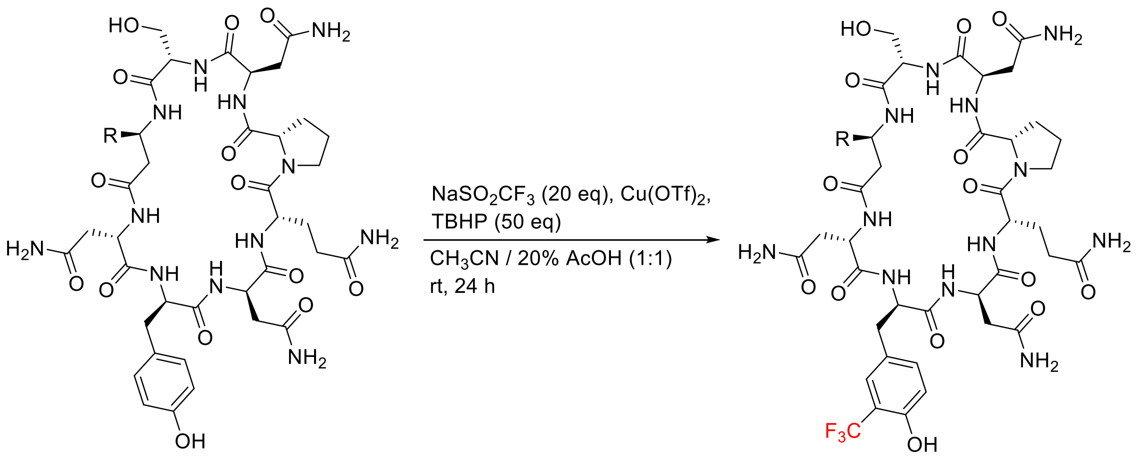

Bacterially produced iturin A - mixture of C_{14} and C_{15}-iturin A

(10b), ~50% conversion

The late-stage trifluoromethylation of bacterially produced iturin A was performed as described above for the synthetic iturin A2. Iturin A (5 mg, ~4.8 µmol, 1 eq), $NaSO_2CF_3$ (15 mg, 96 µmol, 20 eq) and $Cu(OTf)_2$ (1 mg, 2.8 µmol, 0.6 eq) were placed in a 2 mL Eppendorf tube and were dissolved in degassed 1:1 CH_3CN/ 20% AcOH (1 mL). A solution of *tert*-butyl hydroperoxide (70% in H_2O, 33 µL, 0.24 mmol, 50 eq) in degassed 1:1 CH_3CN/ 20% AcOH (0.3 mL) was added dropwise to the initial mixture. The reaction was left for 24 hours at room temperature without stirring and the mixture was added to a Waters Sep-Pak C18 cartridge activated with 2 mL MeOH and 10 mL H_2O. The crude mixture was added to the cartridge, washed with 10 mL of H_2O and the products were eluted with 10 mL MeOH. The obtained solution was analysed via LC-MS, which showed the presence of four compounds: C_{14}-iturin A, C_{15}-iturin A, C_{14}-CF_3-iturin A, and C_{15}-CF_3-iturin A (**Fig. S2**). The products were not purified, but LC-MS analysis suggests a conversion to the trifluoromethylated lipopeptides of approximately 50%.

CF_3-C_{14}-iturin A: **HRMS** (ESI-TOF) calculated for $C_{49}H_{74}F_3N_{12}O_{14}^+$ $[M+H]^+$: 1111.5394, found: 1111.5390.

CF_3-C_{15}-iturin A: **HRMS** (ESI-TOF) calculated for $C_{50}H_{76}F_3N_{12}O_{14}^+$ $[M+H]^+$: 1125.5551, found: 1125.5547.

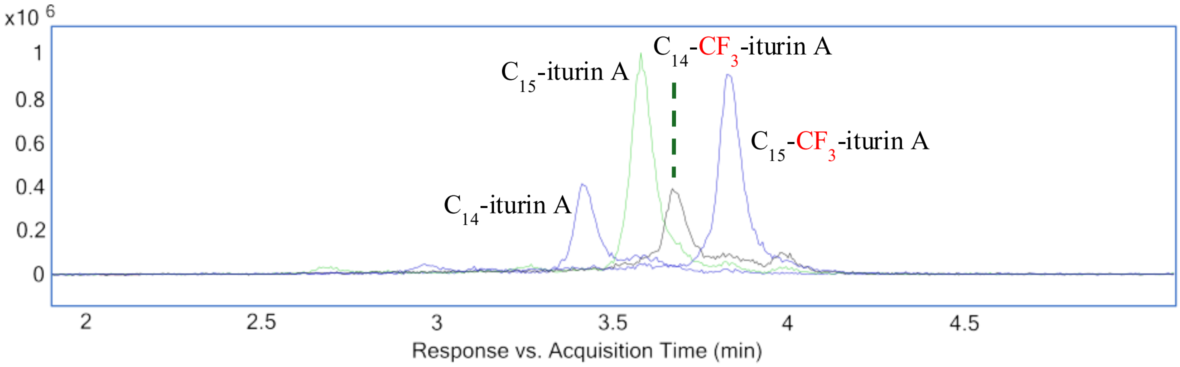

Figure S2: LC-MS analysis (ESI-TOF) of the late-stage trifluoromethylation of bacterially produced iturin A.

Antifungal Susceptibility Testing

The Minimum Inhibitory Concentration (MIC) was calculated for the obtained lipopeptides against *Candida albicans* and *Fusarium graminearum*, according to the CLSI guidelines M27-A2[9] and M38-A[10], respectively. Stock solutions of the lipopeptides were prepared in DMSO and diluted to the desired concentration of 256 µM with RPMI 1640 medium. The maximum

concentration of DMSO that was present in the assays (2.5% v/v) was determined to not inhibit fungal growth in control experiments.

C. albicans was grown in Saboraud dextrose agar for 24 hours at 37 ^{o}C. Five colonies were picked and suspended in 5 mL of saline solution (0.85% w/v) and the OD_{600} was adjusted to ~ 0.1, using an Epoch Microplate Spectrophotometer (BioTek). The suspension was vortexed for 15 seconds, diluted 1:100 with saline and 1:20 with RPMI 1640 broth medium (pH=7) and was used as the inoculum.

F. graminearum was grown in Saboraud dextrose agar for 2 days at 35° C and for 5 days at 28 ^{o}C. The resulting fungal culture was blended with 200 mL of sterile water and 1 mL of the resulting heterogeneous mixture was suspended in 5 mL of saline solution (0.85% w/v). The OD_{600} was adjusted to ~ 0.15. The suspension was vortexed for 30 seconds, diluted 1:50 with RPMI 1640 broth medium (pH=7) and was used as the inoculum.

The assays were performed in sterile flat-bottom polystyrene 96-well plates (Greiner), using the broth microdilution method. For *C. albicans* the 96-well plates were incubated at 35 ^{o}C for 24 h with no agitation, while for *F. graminearum* they were incubated for 28 ^{o}C for 48 h with no agitation. The well plates were then inspected visually for the presence or absence of fungal growth. The Minimum Inhibitory Concentration (MIC) was defined as the lowest concentration of lipopeptides where complete inhibition of fungal growth was observed. The MIC calculations reflect triplicate experiments.

NMR Spectra

^{1}H NMR (400 MHz, $CDCl_3$) Methyl (*E*)-12,12,12-trifluorododec-9-enoate **(1)**

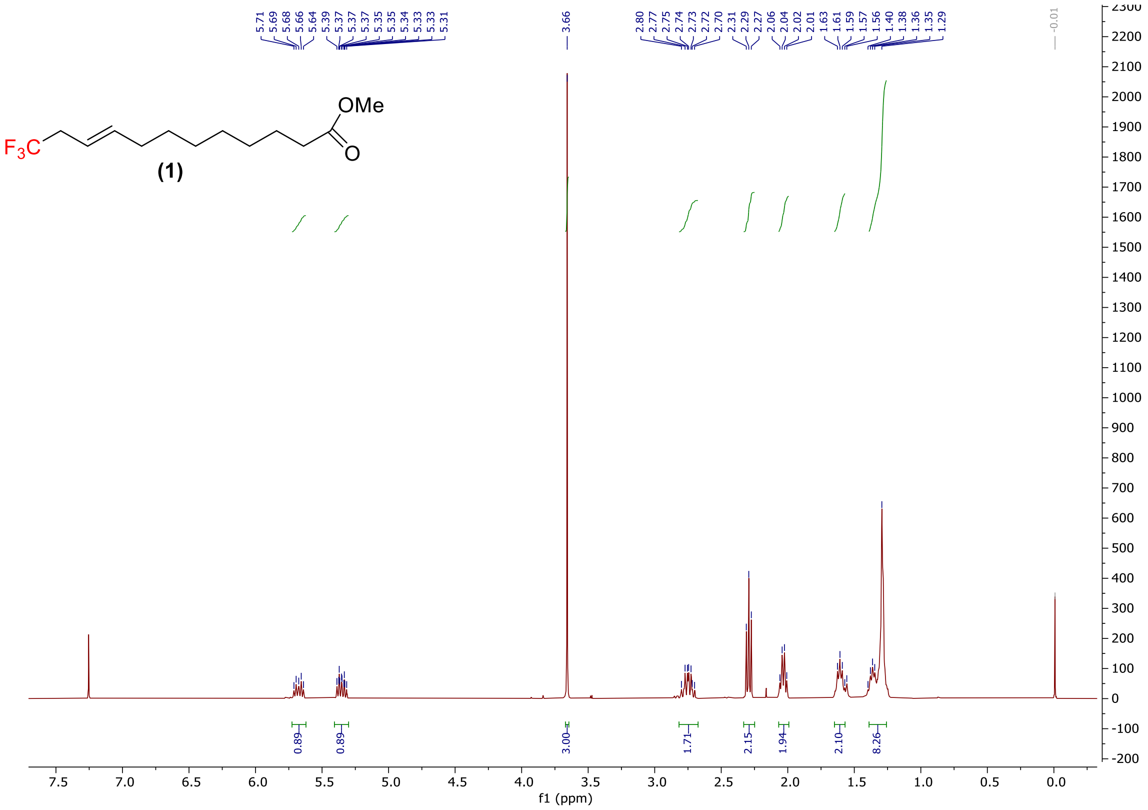

^{13}C NMR (101 MHz, $CDCl_3$) Methyl (*E*)-12,12,12-trifluorododec-9-enoate **(1)**

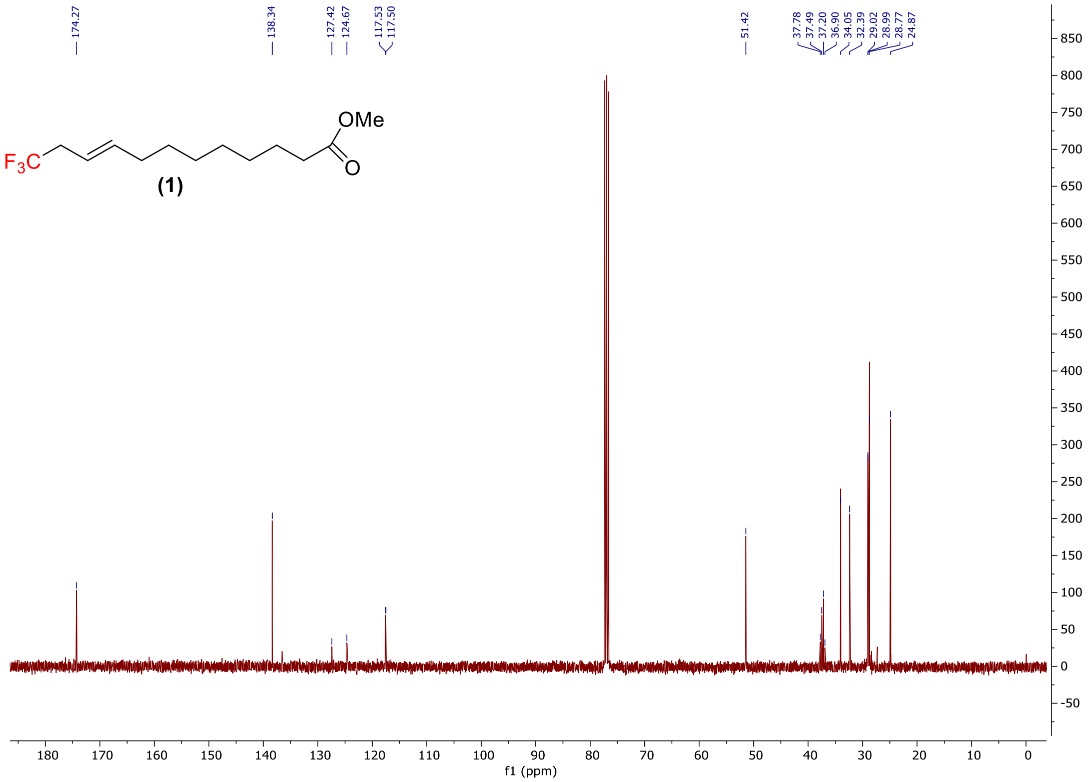

^{19}F NMR (376 MHz, $CDCl_3$) Methyl (*E*)-12,12,12-trifluorododec-9-enoate **(1)**

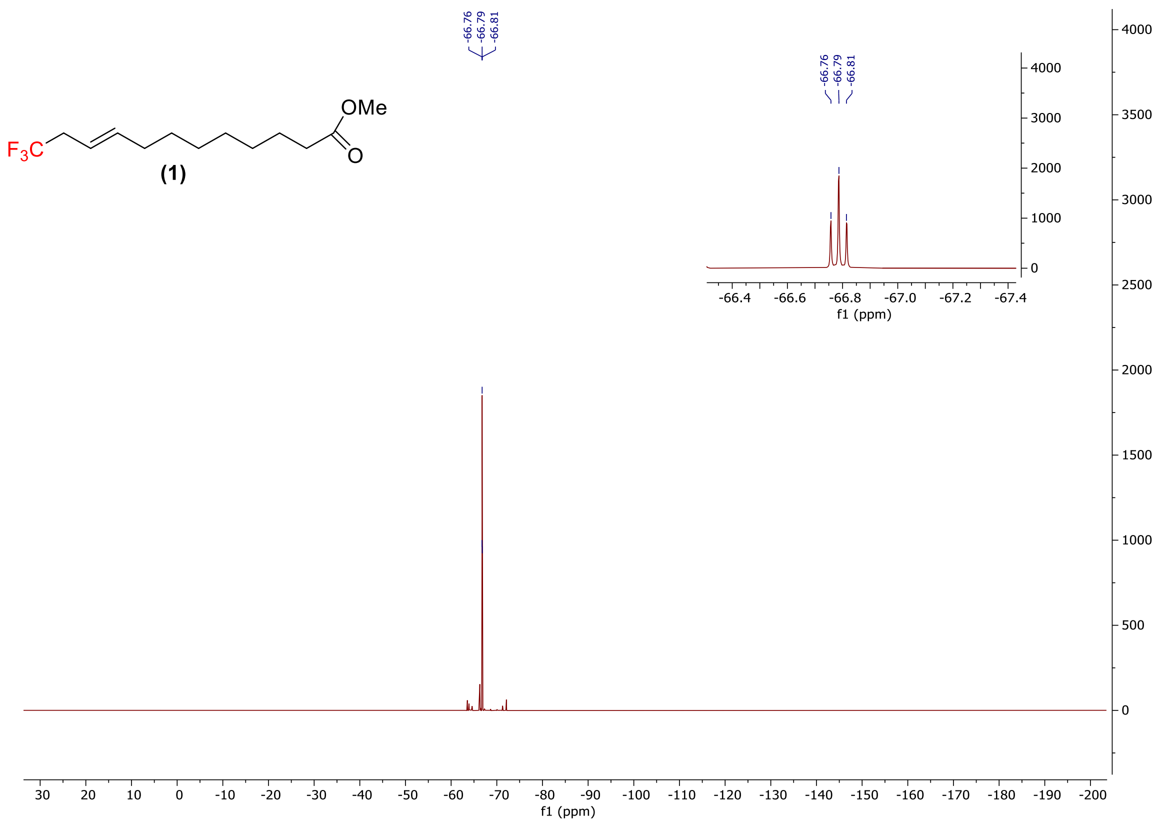

^{1}H NMR (400 MHz, $CDCl_3$) Methyl 12,12,12-trifluorododecanoate **(2)**

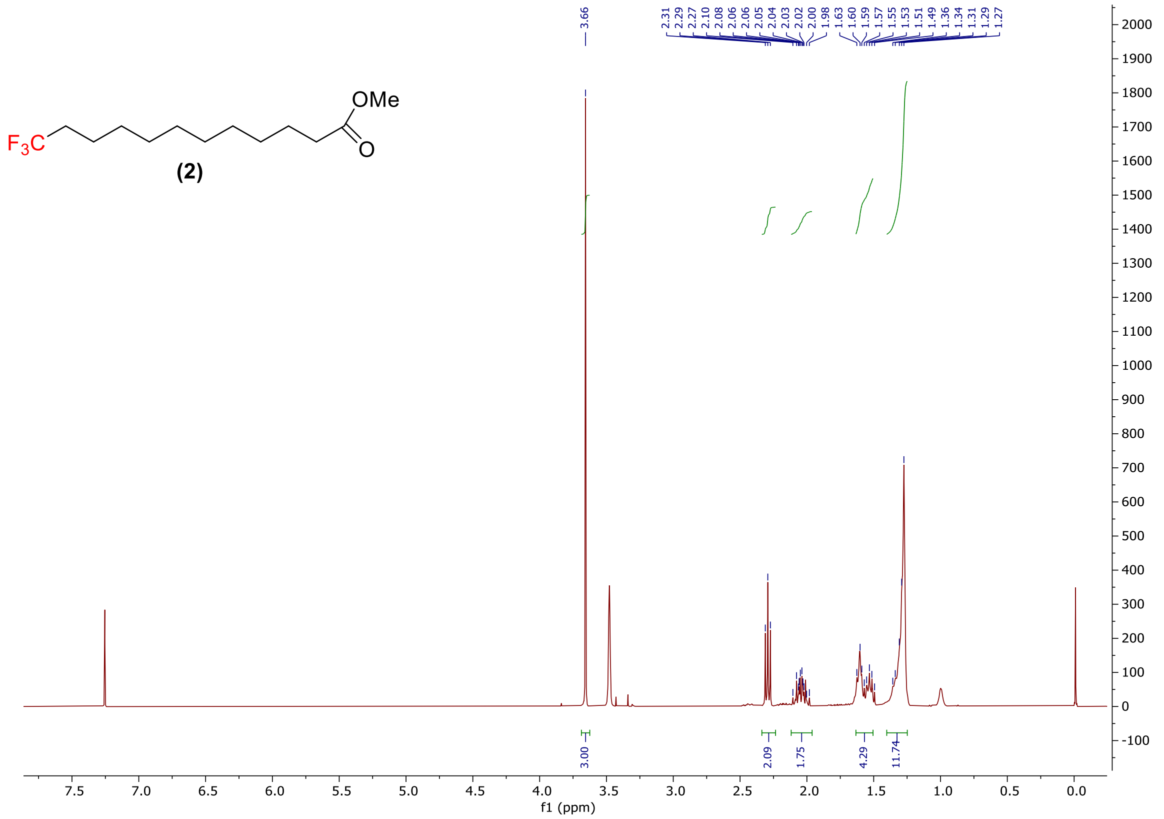

^{13}C NMR (101 MHz, $CDCl_3$) Methyl 12,12,12-trifluorododecanoate **(2)**

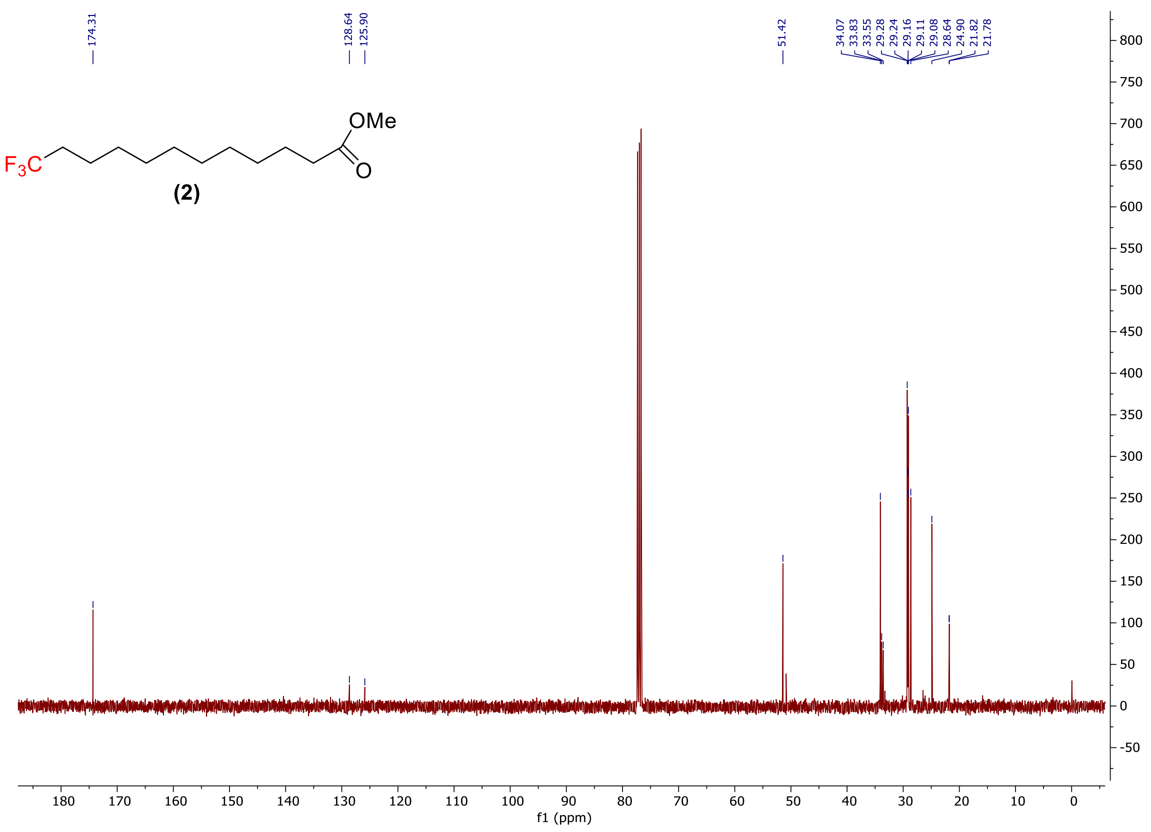

^{19}F NMR (376 MHz, $CDCl_3$) Methyl 12,12,12-trifluorododecanoate **(2)**

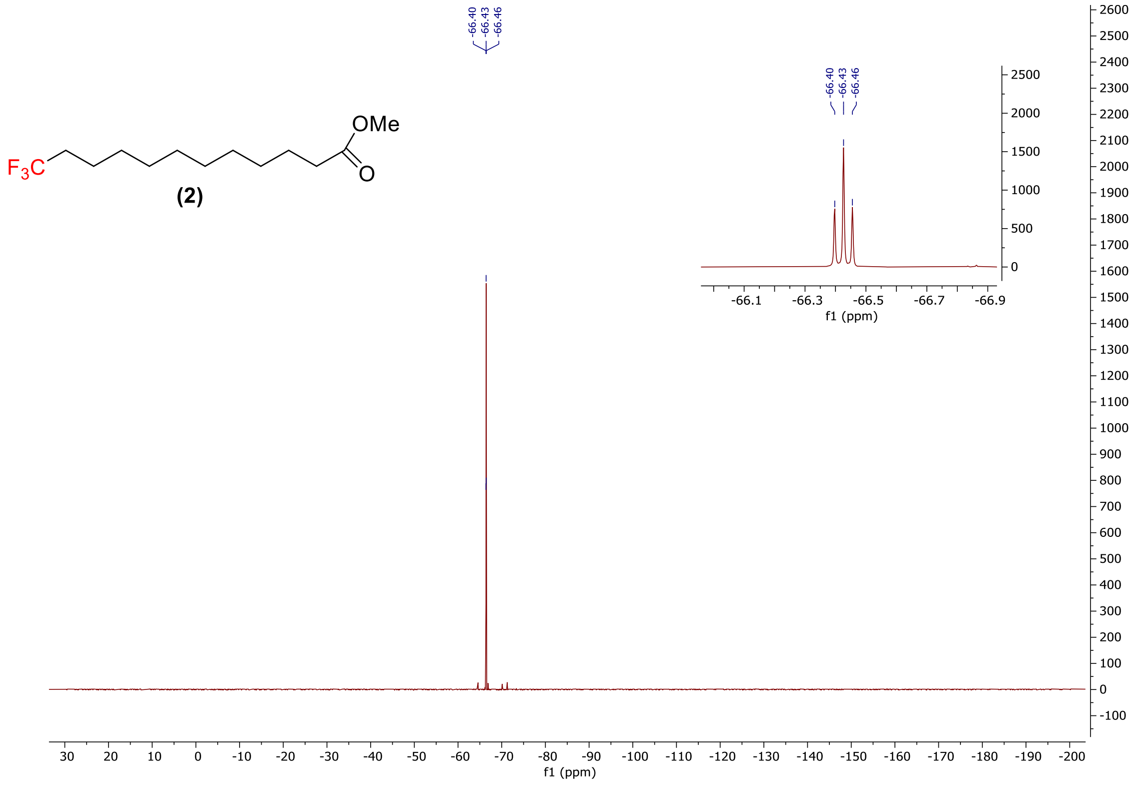

^{1}H NMR (400 MHz, $CDCl_3$) 12,12,12-Trifluorododecan-1-ol **(3)**

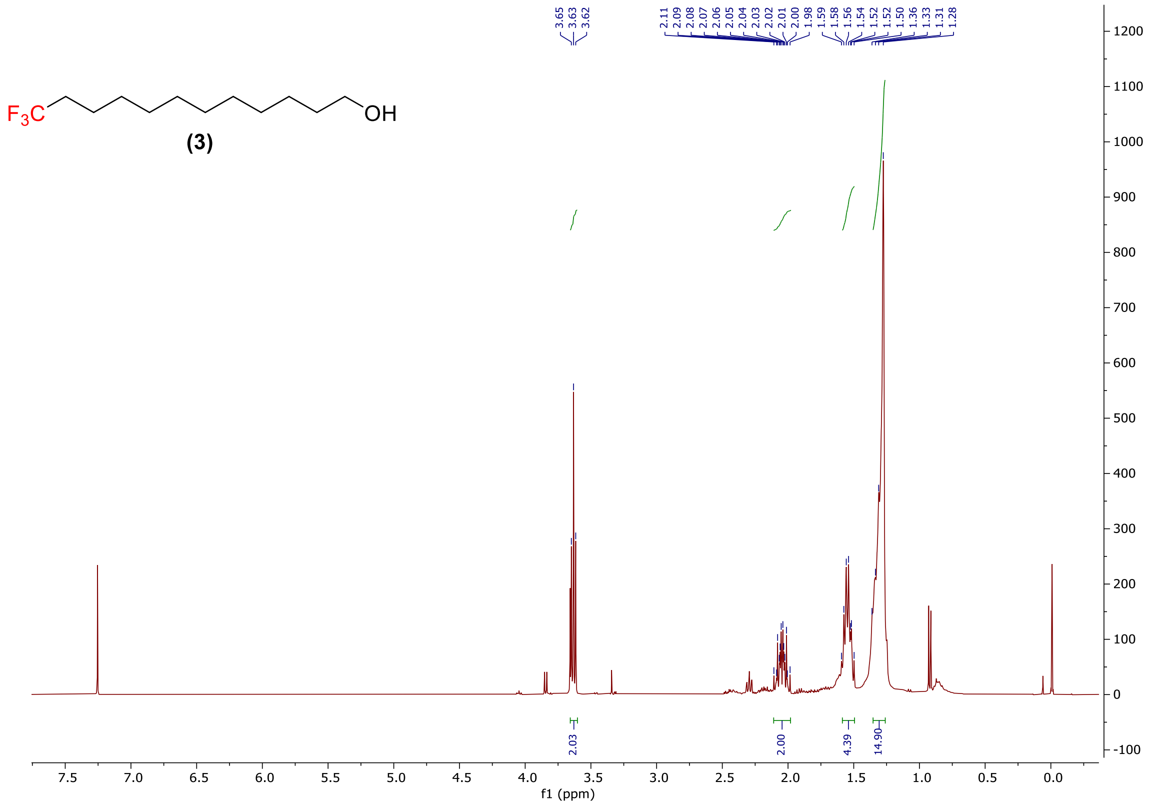

^{13}C NMR (101 MHz, $CDCl_3$) 12,12,12-Trifluorododecan-1-ol **(3)**

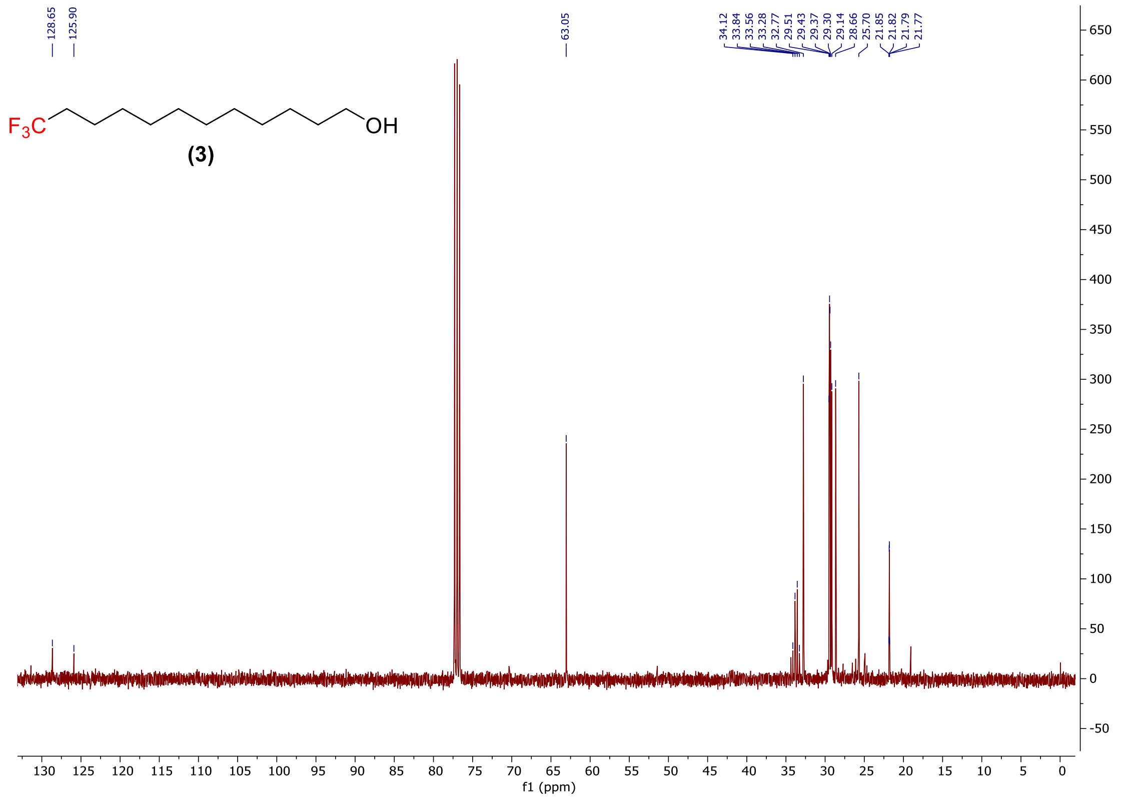

^{19}F NMR (376 MHz, $CDCl_3$) 12,12,12-Trifluorododecan-1-ol **(3)**

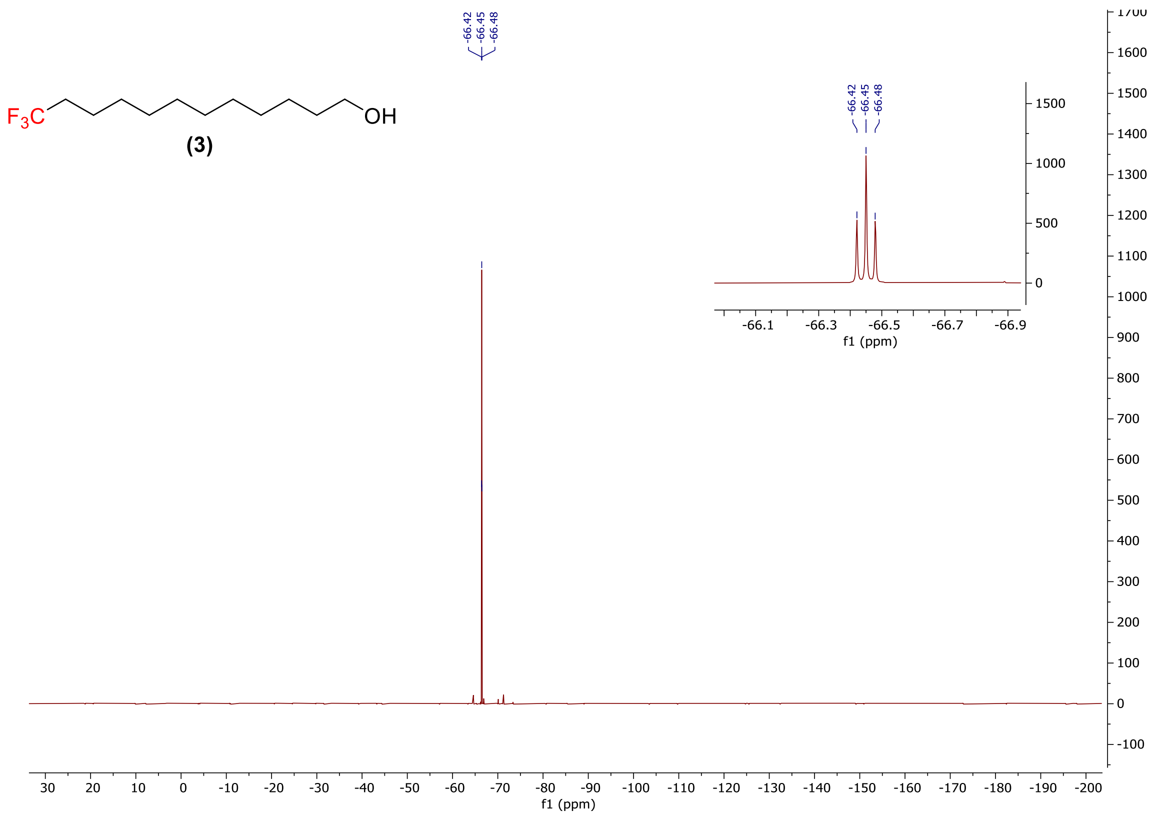

^{1}H NMR (400 MHz, $CDCl_3$) Ethyl (*E*)-14,14,14-trifluorotetradec-2-enoate **(4)**

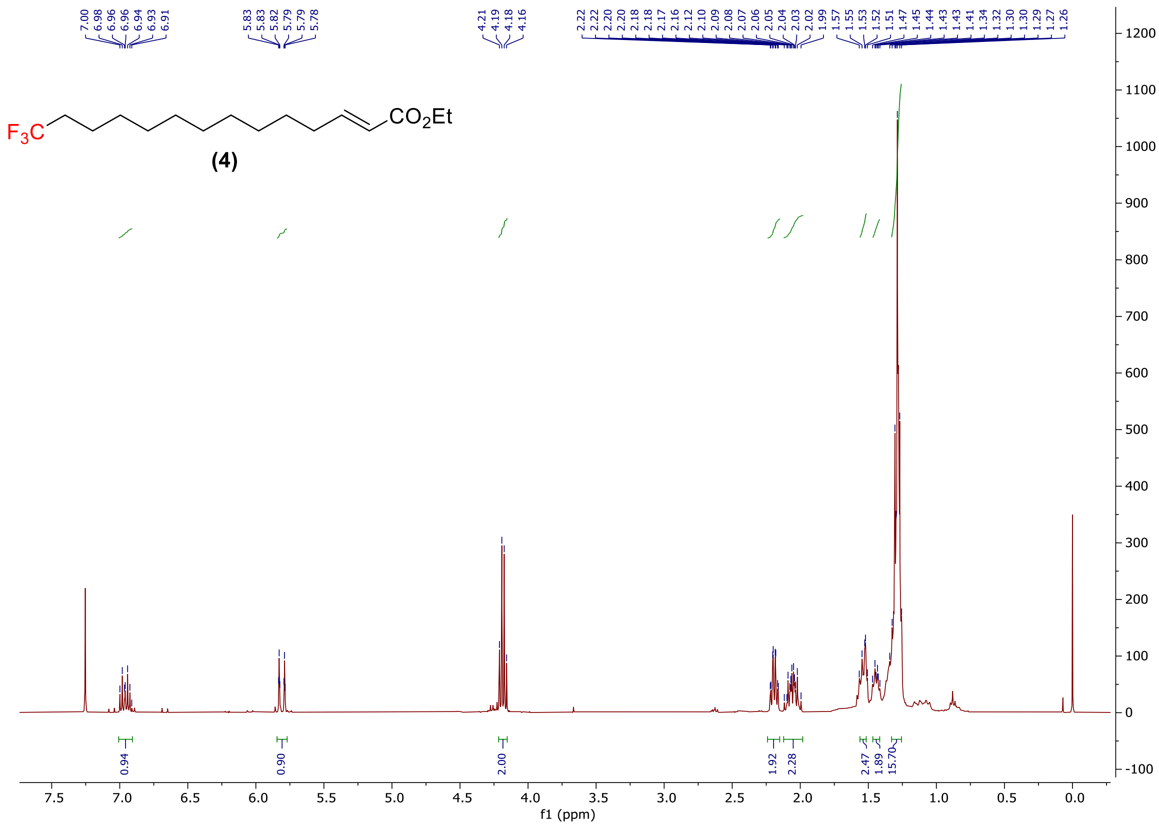

^{13}C NMR (101 MHz, $CDCl_3$) Ethyl (*E*)-14,14,14-trifluorotetradec-2-enoate **(4)**

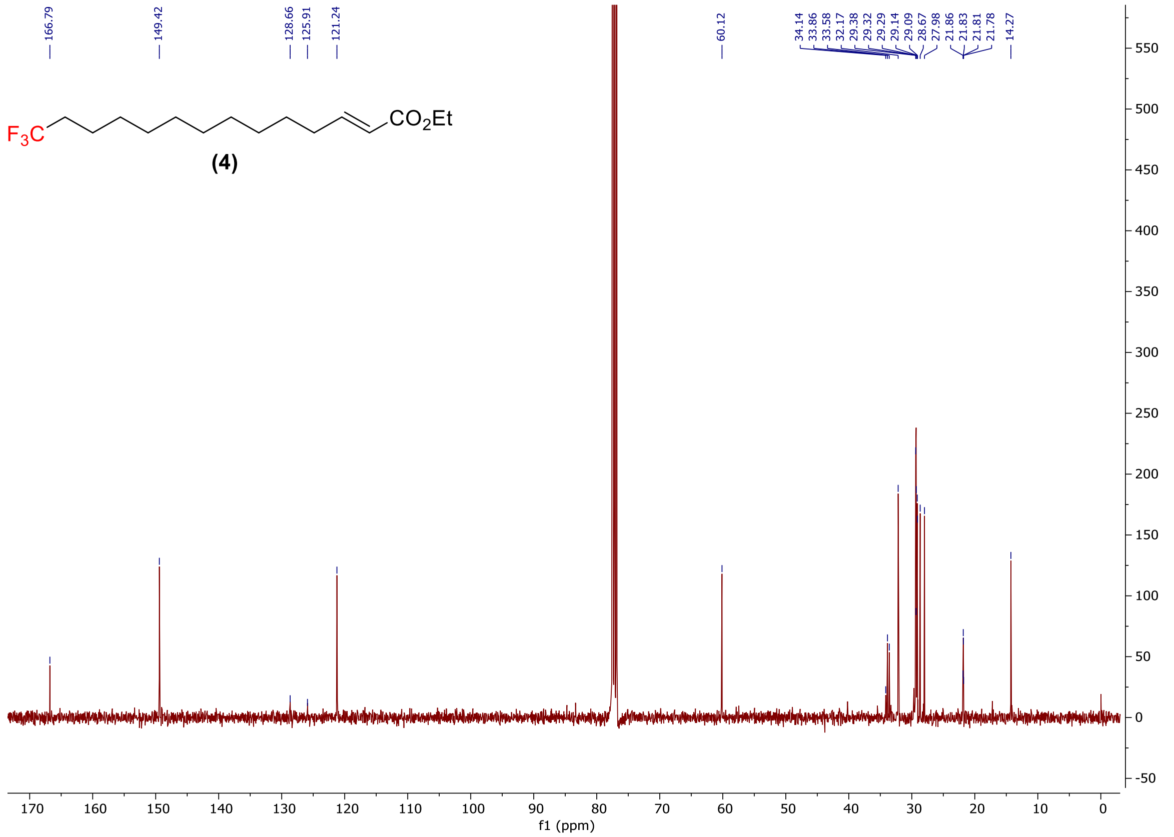

^{19}F NMR (376 MHz, $CDCl_3$) Ethyl (*E*)-14,14,14-trifluorotetradec-2-enoate **(4)**

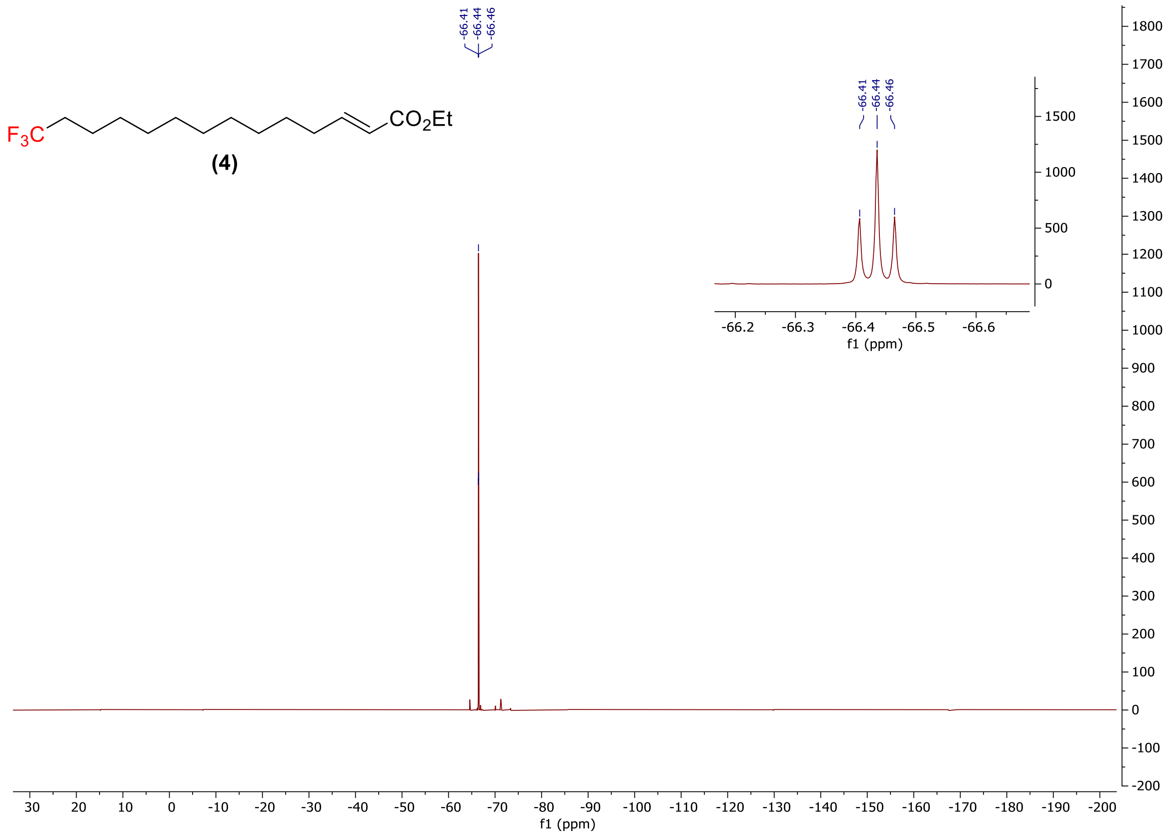

^{1}H NMR (400 MHz, $CDCl_3$) Ethyl (*R*)-3-(benzyl((*R*)-1-phenylethyl)amino)-14,14,14-trifluorotetradecanoate **(5)**

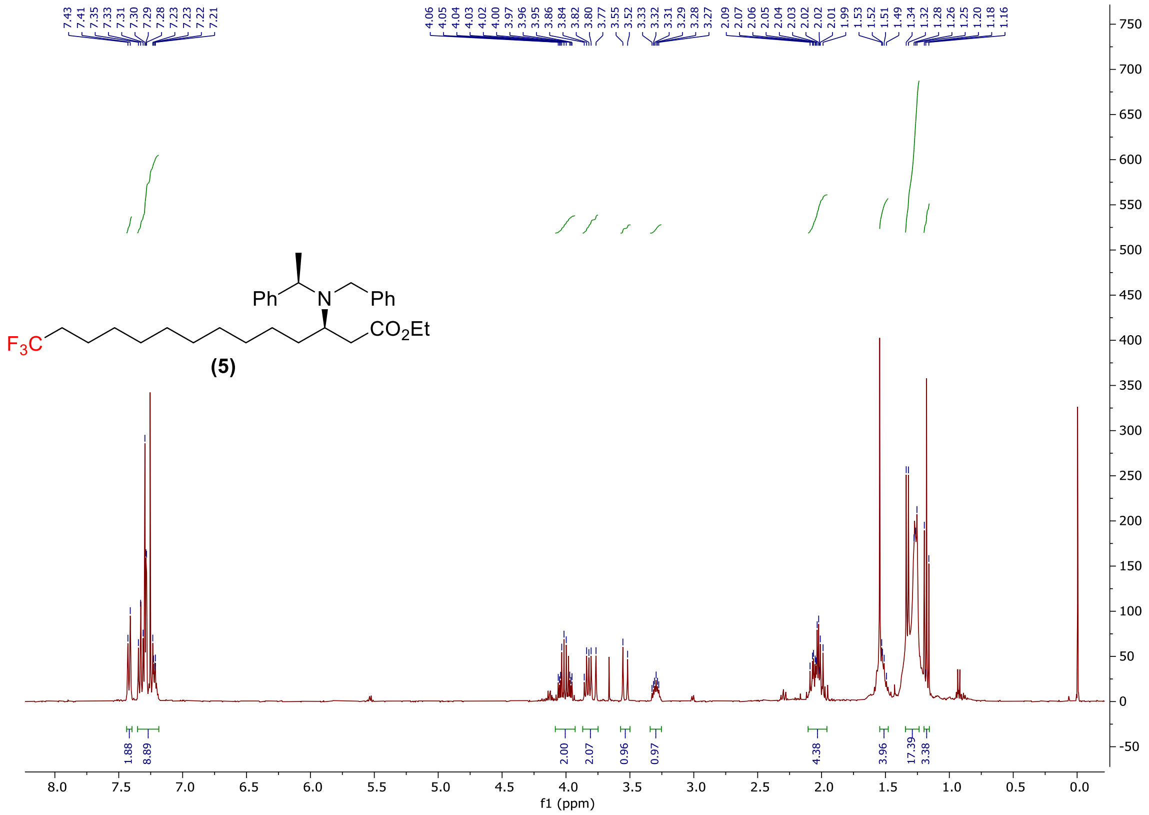

^{13}C NMR (101 MHz, $CDCl_3$) Ethyl (*R*)-3-(benzyl((*R*)-1-phenylethyl)amino)-14,14,14-trifluorotetradecanoate **(5)**

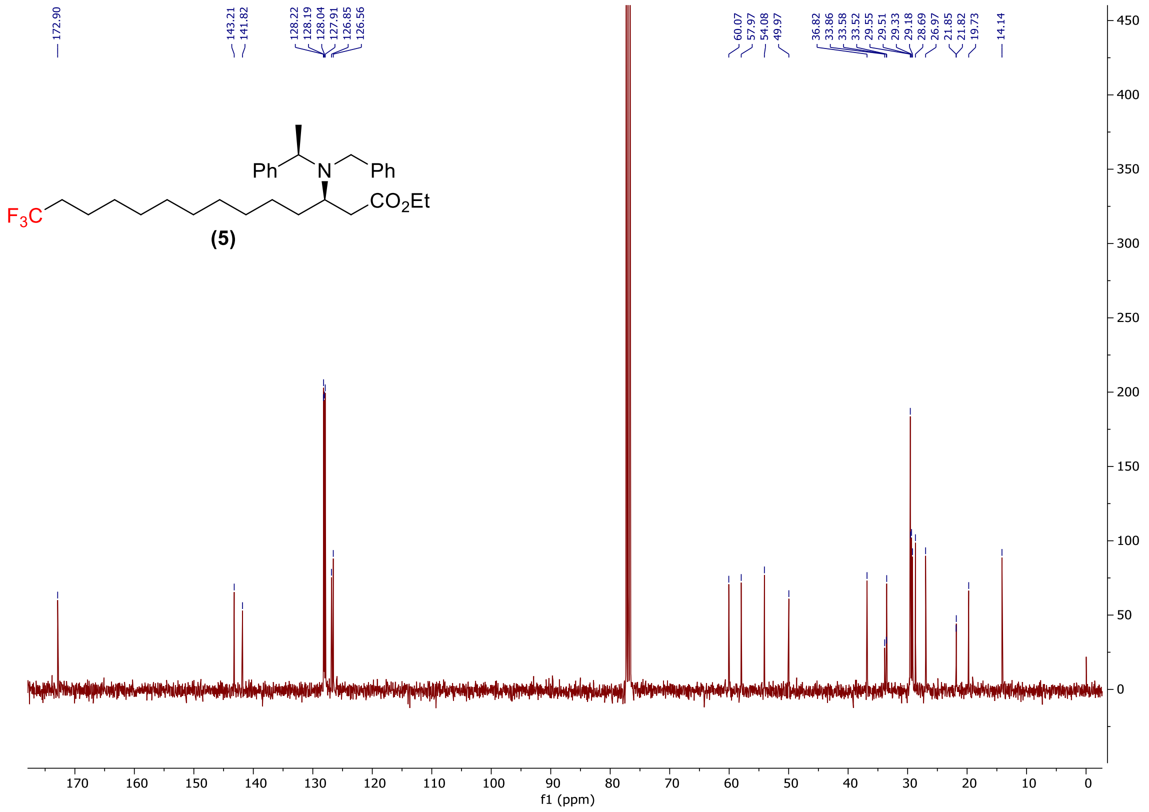

^{19}F NMR (376 MHz, $CDCl_3$) Ethyl (*R*)-3-(benzyl((*R*)-1-phenylethyl)amino)-14,14,14-trifluorotetradecanoate **(5)**

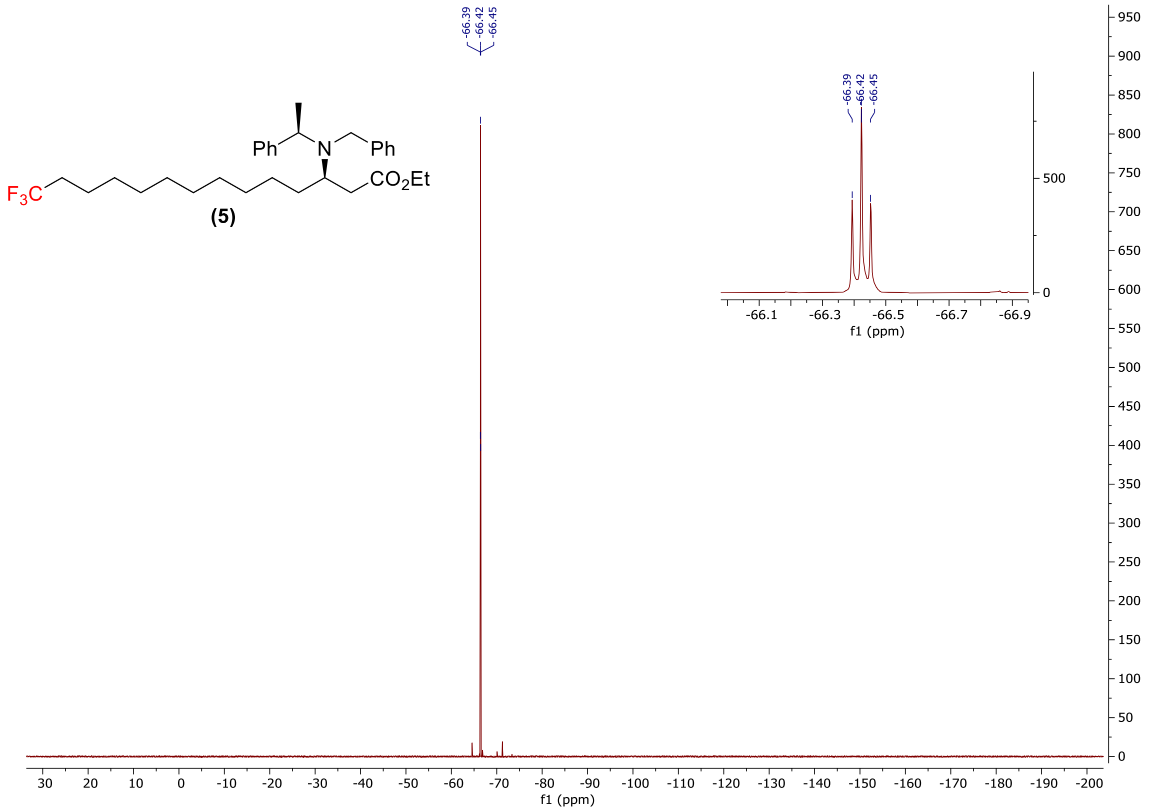

^{1}H NMR (500 MHz, $CDCl_3$) (*R*)-3-((((9*H*-Fluoren-9-yl)methoxy)carbonyl)amino)-14,14,14-trifluorotetradecanoic acid **(6)**

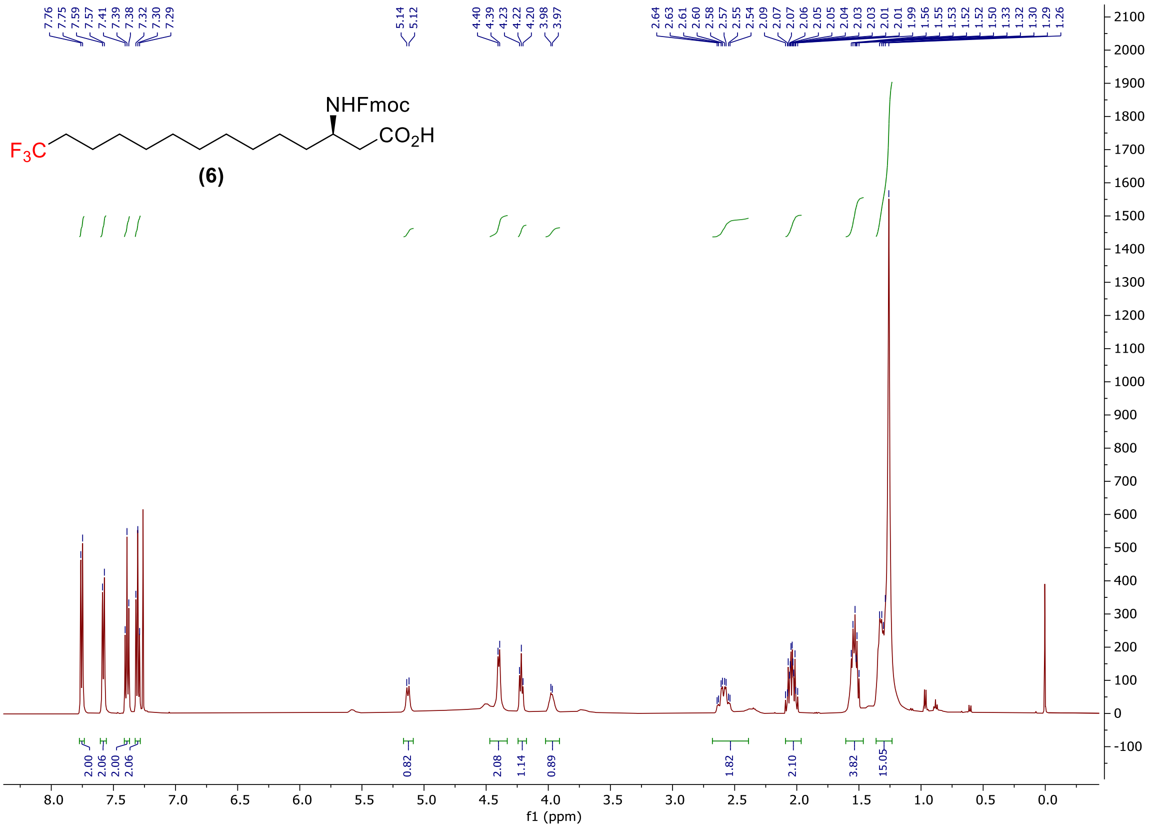

^{13}C NMR (125 MHz, $CDCl_3$) (*R*)-3-((((9*H*-Fluoren-9-yl)methoxy)carbonyl)amino)-14,14,14-trifluorotetradecanoic acid **(6)**

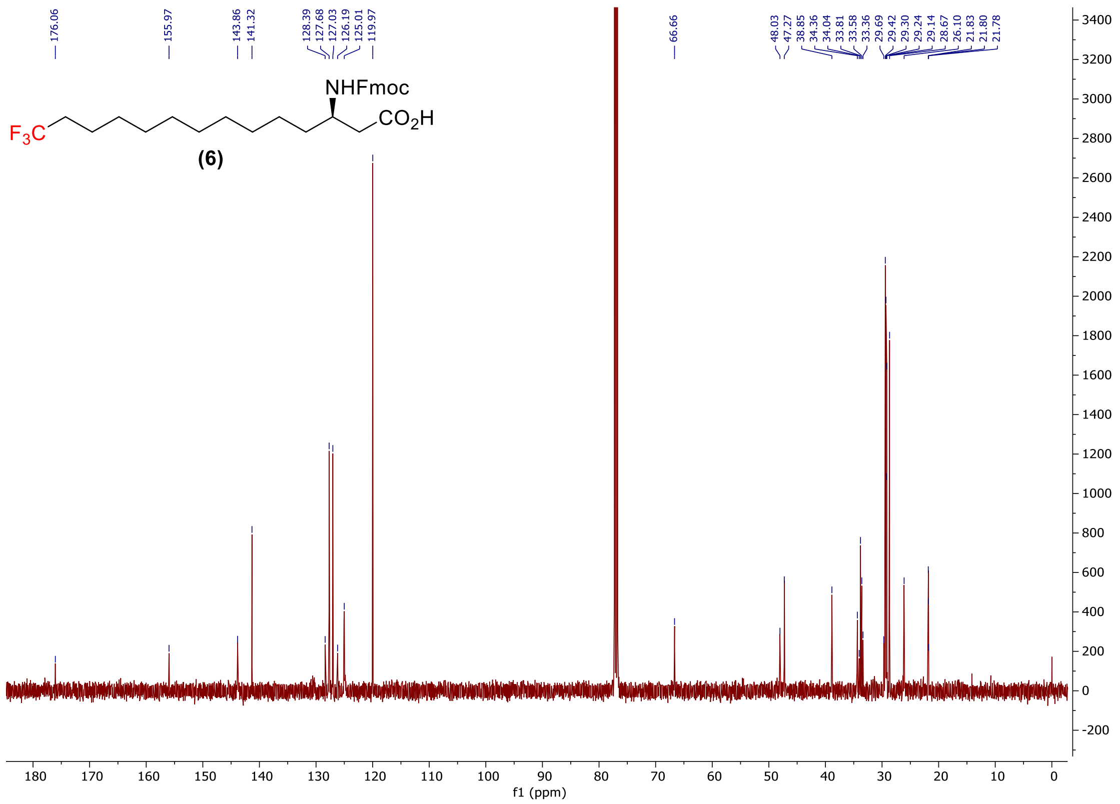

^{19}F NMR (470 MHz, $CDCl_3$) (*R*)-3-((((9*H*-Fluoren-9-yl)methoxy)carbonyl)amino)-14,14,14-trifluorotetradecanoic acid **(6)**

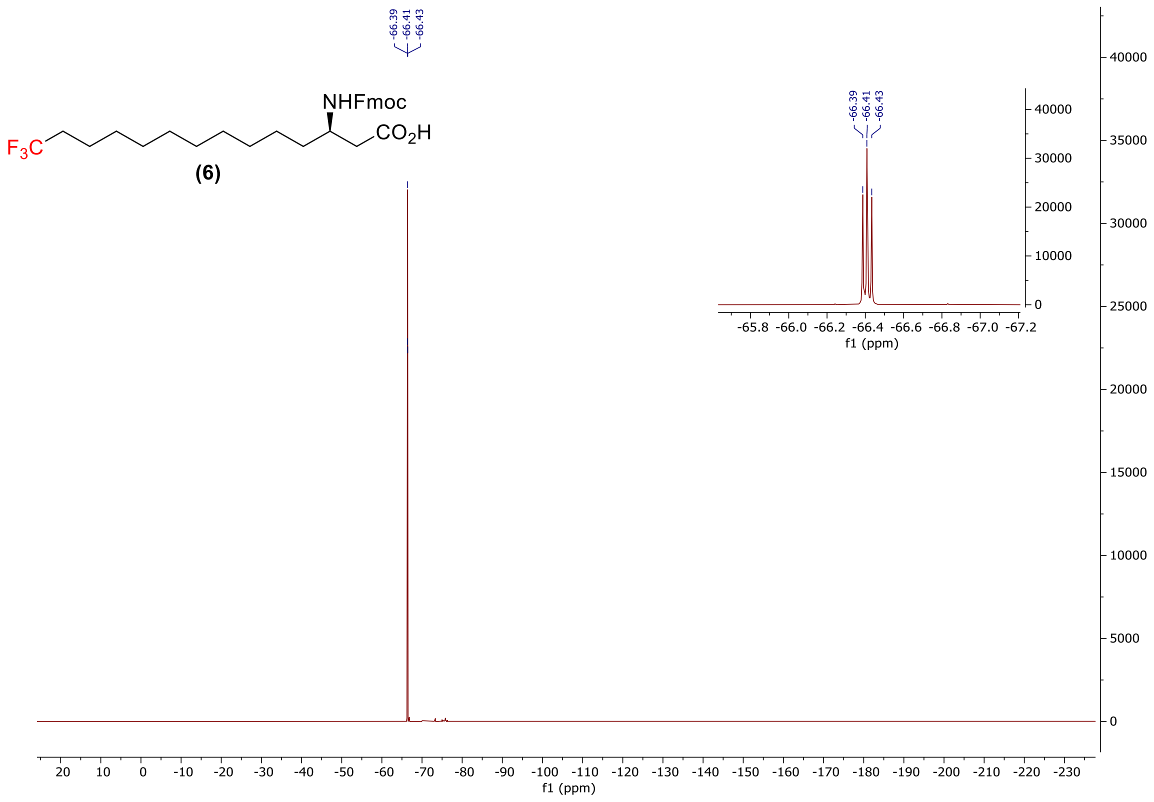

^{1}H NMR (400 MHz, d_6-DMSO) (*R*)-2-((((9*H*-Fluoren-9-yl)methoxy)carbonyl)amino)-3-(4-hydroxy-3-(trifluoromethyl)phenyl)propanoic acid **(7)**

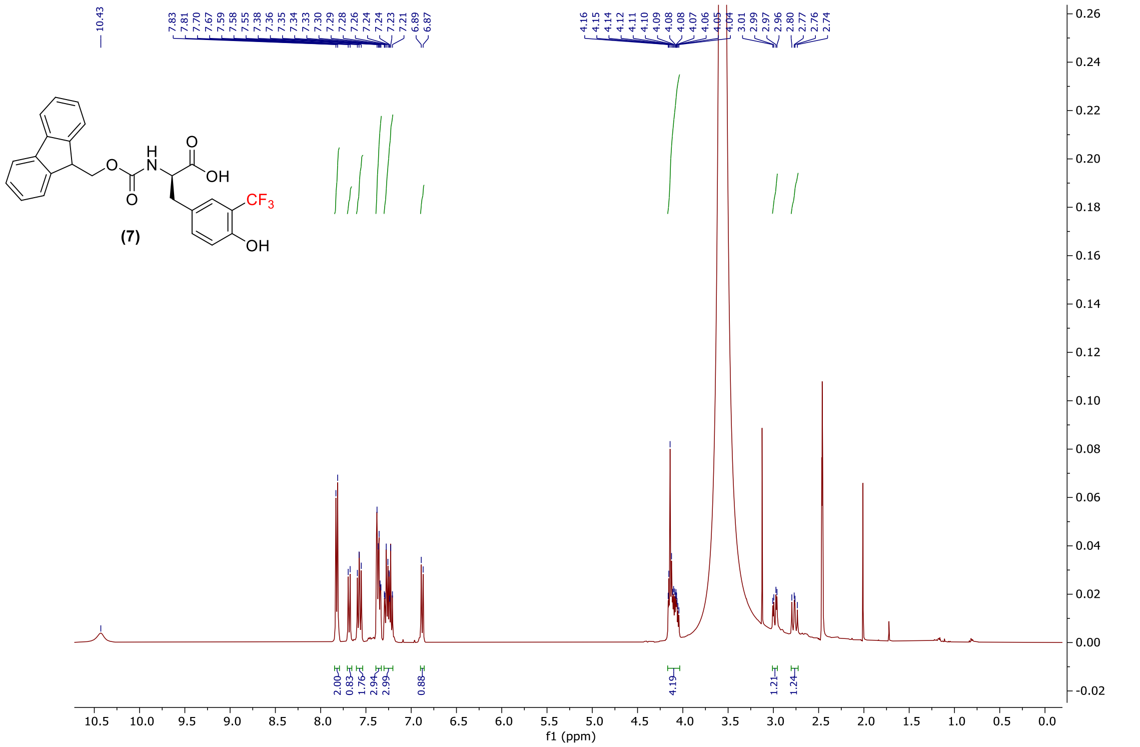

^{13}C NMR (101 MHz, d_6-DMSO) (*R*)-2-((((9*H*-Fluoren-9-yl)methoxy)carbonyl)amino)-3-(4-hydroxy-3-(trifluoromethyl)phenyl)propanoic acid **(7)**

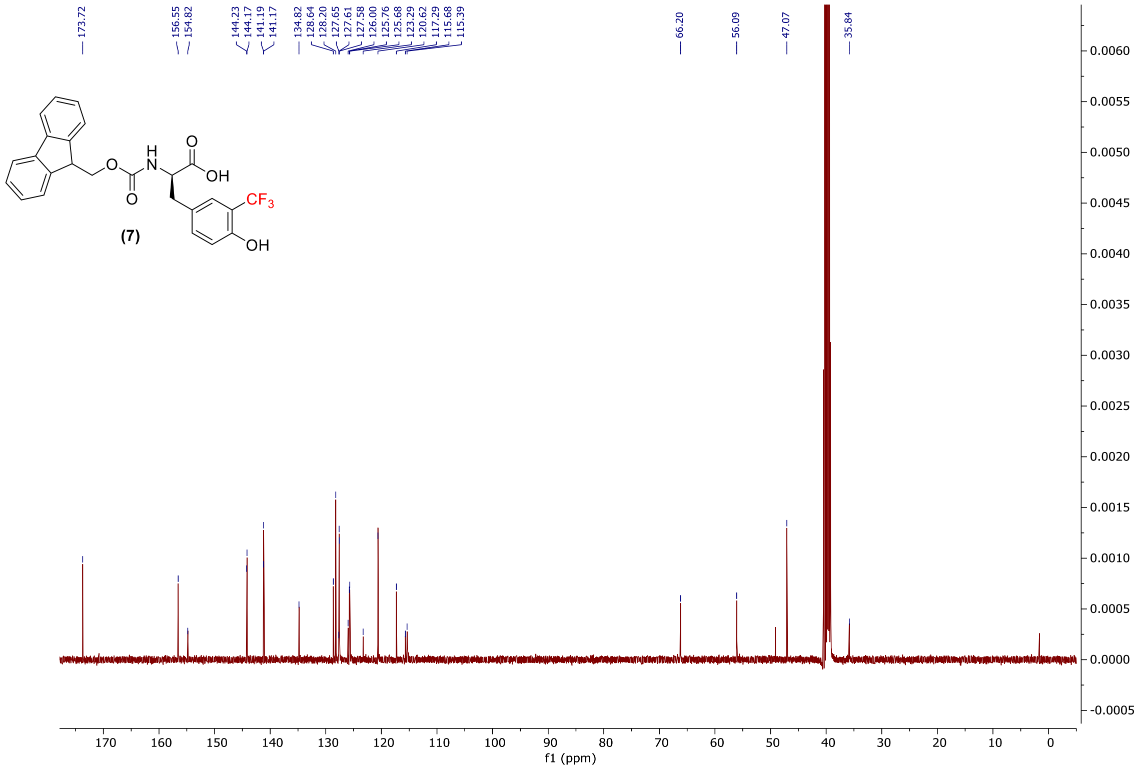

^{19}F NMR (376 MHz, d_6-DMSO) (*R*)-2-((((9*H*-Fluoren-9-yl)methoxy)carbonyl)amino)-3-(4-hydroxy-3-(trifluoromethyl)phenyl)propanoic acid **(7)**

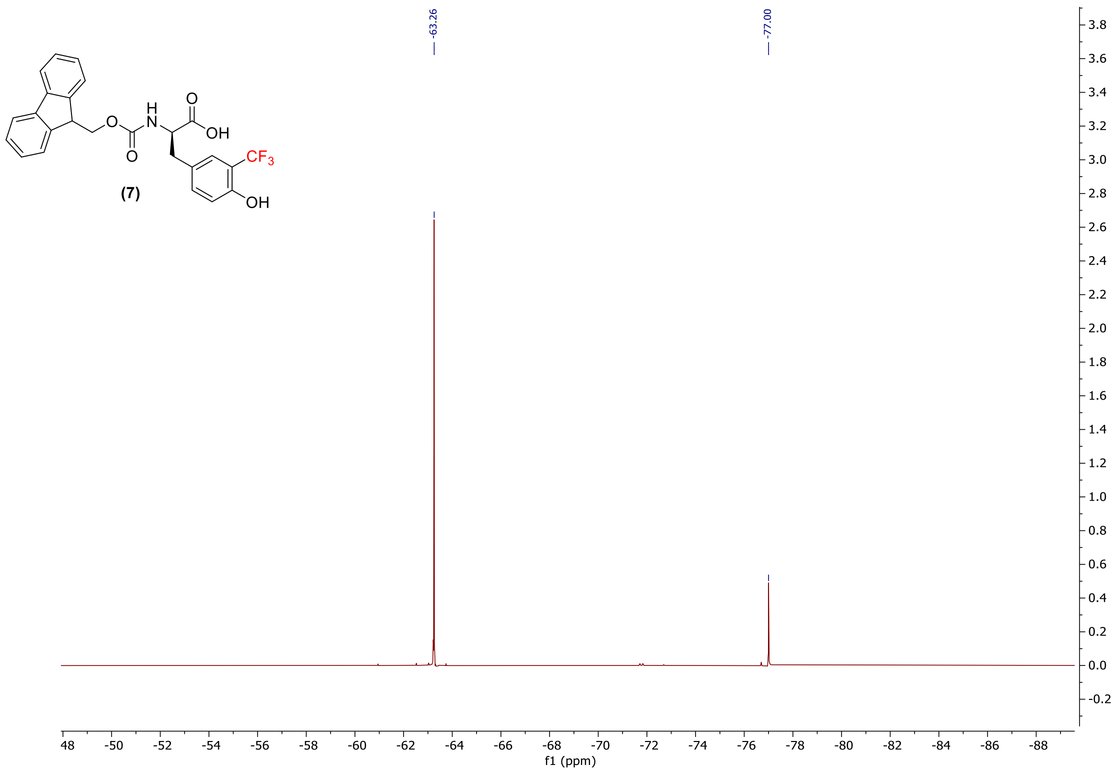

^{1}H NMR (400 MHz, d_6-DMSO) (*R*)-2-((((9*H*-Fluoren-9-yl)methoxy)carbonyl)amino)-3-(4-hydroxy-3,5-bis(trifluoromethyl)phenyl)propanoic acid (**8**)

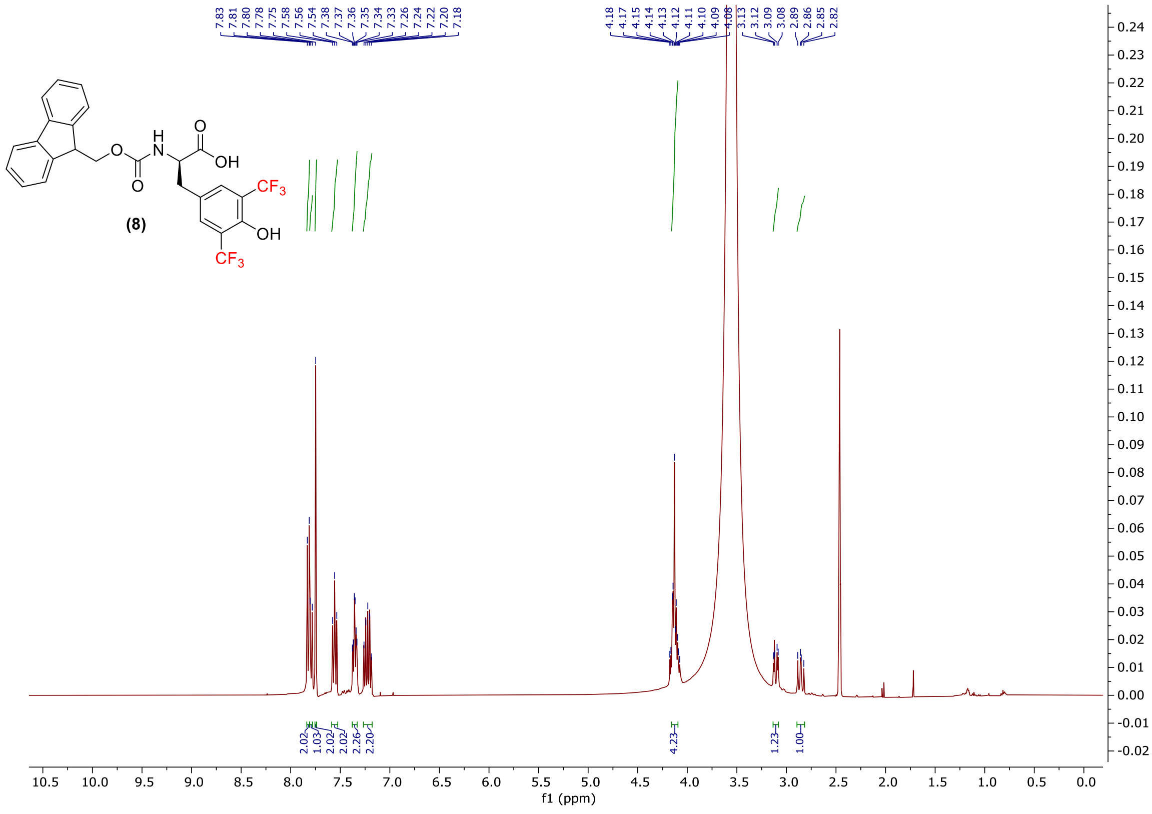

^{13}C NMR (101 MHz, d_6-DMSO) (*R*)-2-((((9*H*-Fluoren-9-yl)methoxy)carbonyl)amino)-3-(4-hydroxy-3,5-bis(trifluoromethyl)phenyl)propanoic acid (**8**)

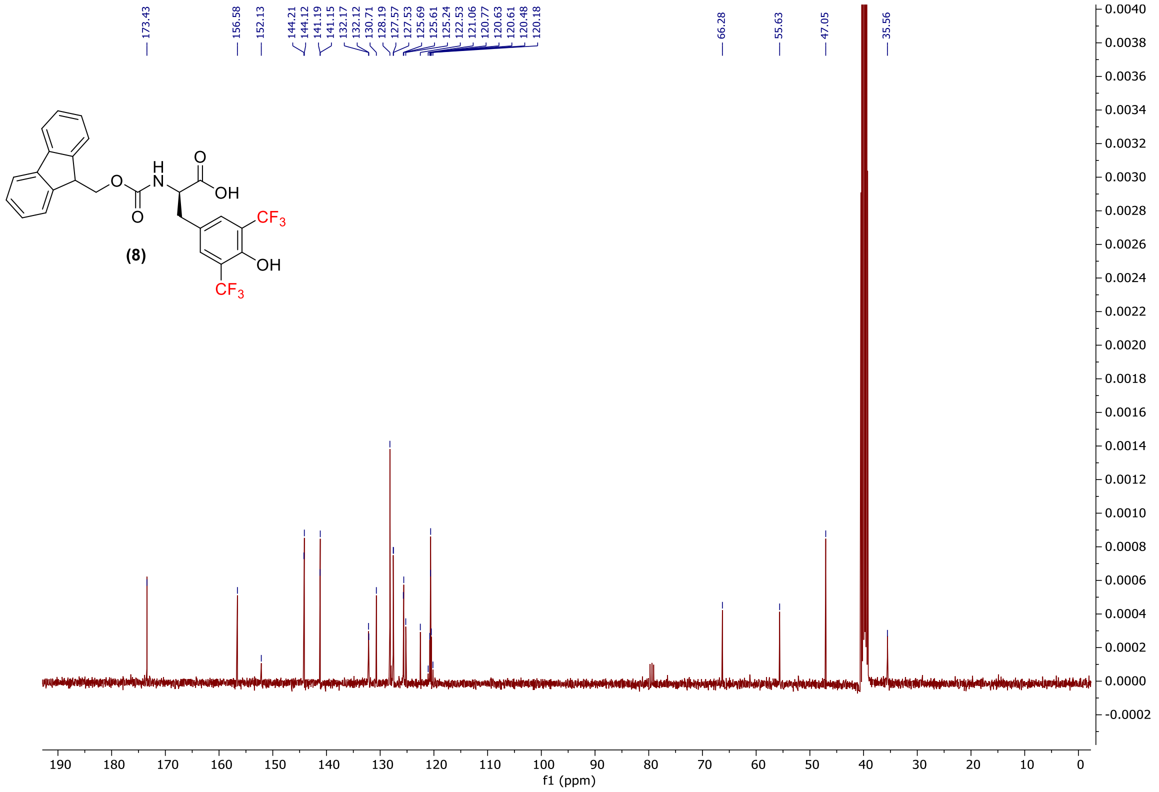

^{19}F NMR (376 MHz, d_6-DMSO) (*R*)-2-((((9*H*-Fluoren-9-yl)methoxy)carbonyl)amino)-3-(4-hydroxy-3,5-bis(trifluoromethyl)phenyl)propanoic acid (**8**)

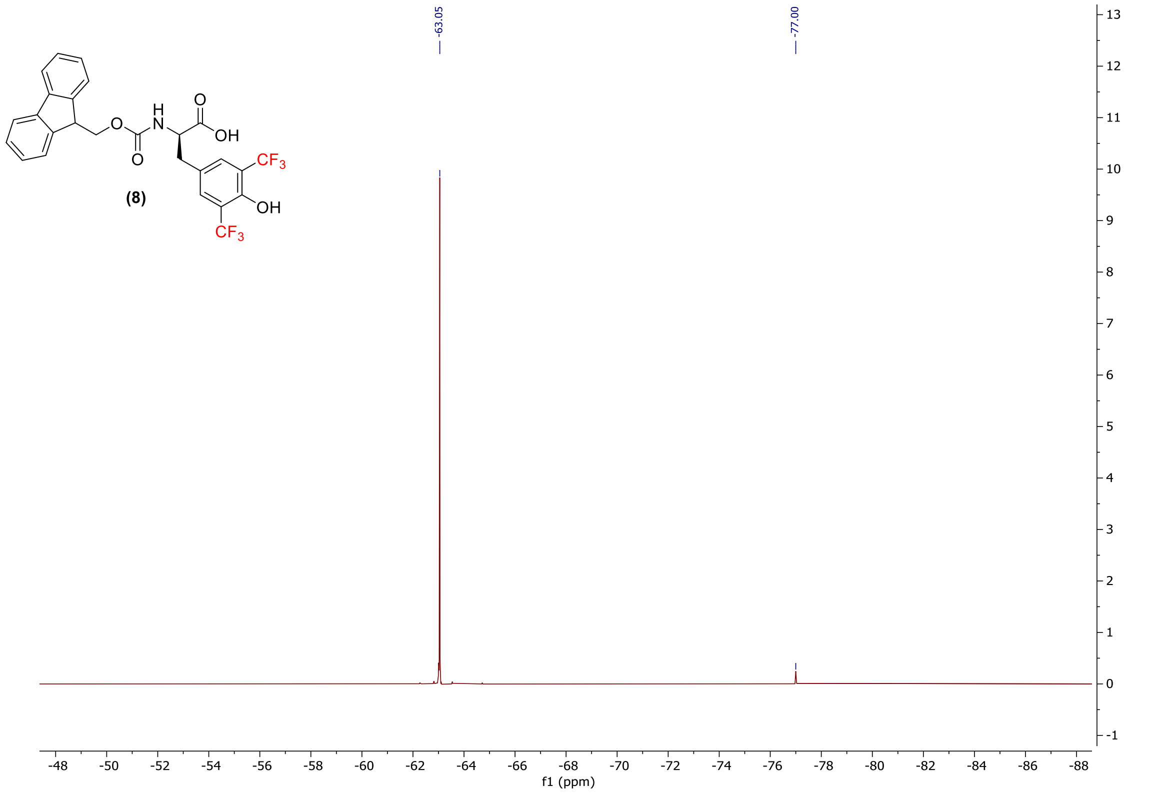

^{19}F NMR (376 MHz, CD_3OD) Alkyl-trifluoromethylated iturin A **(9)**

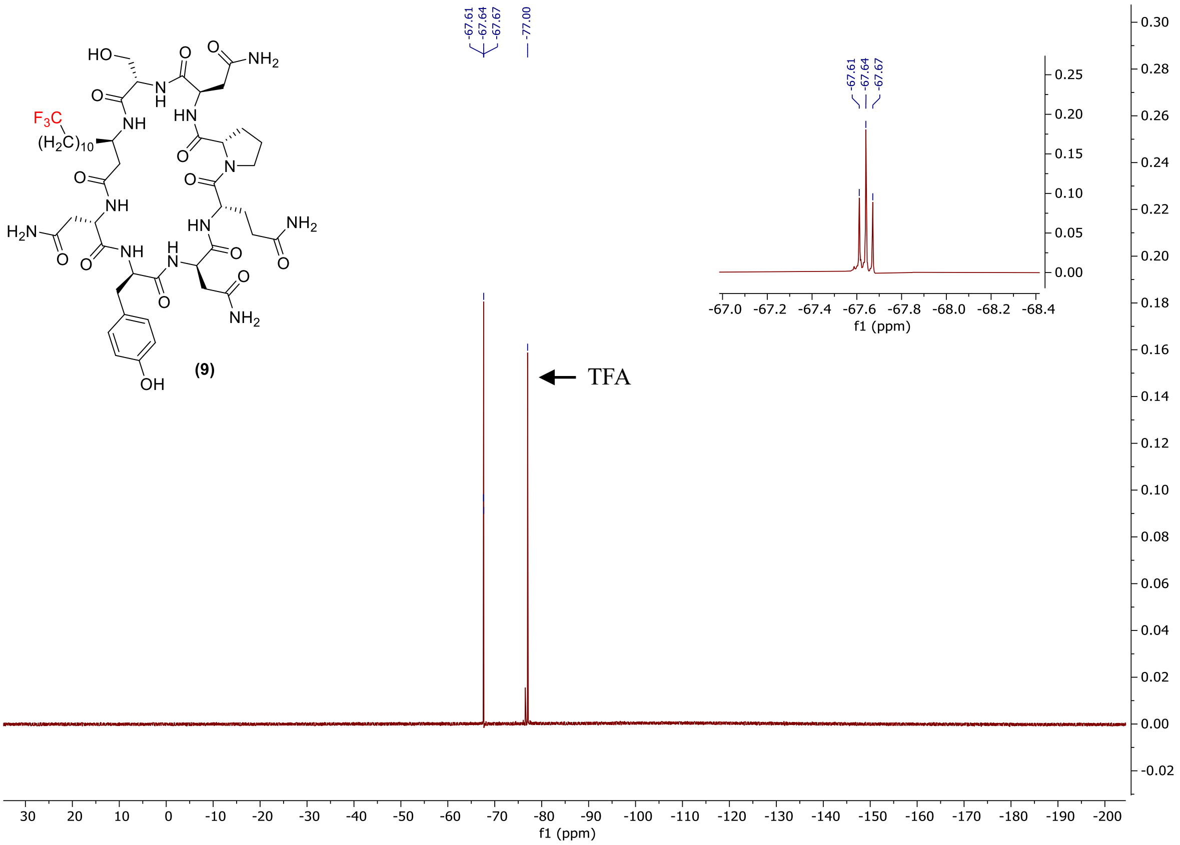

^{19}F NMR (376 MHz, CD_3OD) Trifluoromethylated iturin A **(10)**

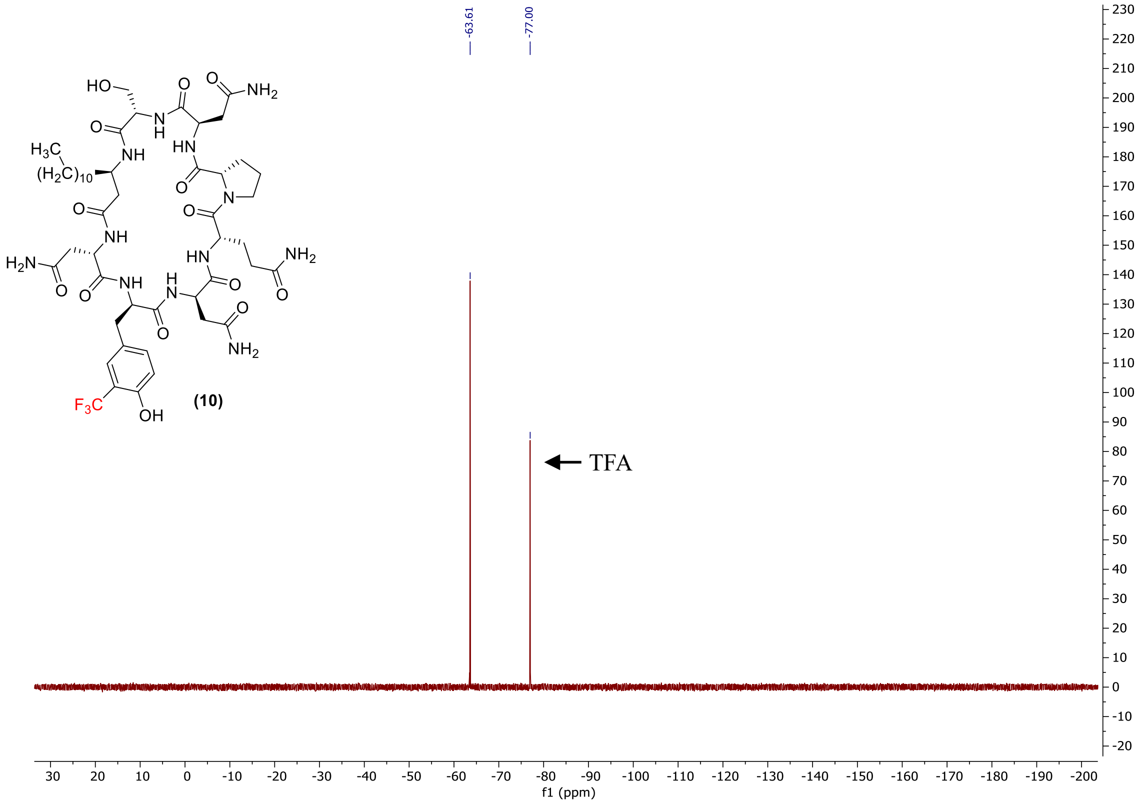

References

[1] A. T. Parsons, S. L. Buchwald, *Angewandte Chemie International Edition* **2011**, *50*, 9120-9123.

[2] C.-H. Chiang, R. Ramu, Y.-J. Tu, *et al.*, *Chemistry – A European Journal* **2013**, *19*, 13680-13691.

[3] W. Zhang, Z. Zou, Y. Wang, *et al.*, *Angewandte Chemie International Edition* **2019**, *58*, 624-627.

[4] S. Ozturk, C. C. Forneris, A. K. L. Nguy, *et al.*, *The Journal of Organic Chemistry* **2018**, *83*, 7309-7317.

[5] P. Karamanis, M. Kiernan, J. Muldoon, *et al.*, *Chemistry – A European Journal*, *n/a*, e01341.

[6] E. Kaiser, R. L. Colescott, C. D. Bossinger, *et al.*, *Analytical Biochemistry* **1970**, *34*, 595-598.

[7] P. Karamanis, J. Muldoon, C. D. Murphy, *et al.*, *Journal of Peptide Science* **2024**, *30*, e3569.

[8] N. K. O'Connor, A. S. Hudson, S. L. Cobb, *et al.*, *Amino Acids* **2014**, *46*, 2745-2752.

[9] Clinical and Laboratory Standards Institute. Reference Method for Broth Dilution Antifungal Susceptibility Testing of Yeasts; Approved Standard – second edition. CLSI document M27-A2, Wayne, Pa., 2002.

[10] Clinical and Laboratory Standards Institute. Reference Method for Broth Dilution Antifungal Susceptibility Testing of Filamentous Fungi; Approved Standard - first edition. CLSI document M38-A, Wayne, Pa., 2002.
